# Mapping global new-onset, worsening, and resolution of diabetes following partial pancreatectomy: a systematic review and meta-analysis

**DOI:** 10.1097/JS9.0000000000000998

**Published:** 2023-12-21

**Authors:** Junlun Wei, Yiran Ou, Jiaoting Chen, Zhicheng Yu, Zhenghao Wang, Ke Wang, Dujiang Yang, Yun Gao, Yong Liu, Jiaye Liu, Xiaofeng Zheng

**Affiliations:** a Department of Endocrinology and Metabolism, Center for Diabetes and Metabolism Research; b Department of Respiratory and Critical Care Medicine, Frontiers Science Center for Disease-Related Molecular Network, Center of Precision Medicine, Precision Medicine Key Laboratory of Sichuan Province; c Laboratory of Thyroid and Parathyroid diseases, Frontiers Science Center for Disease-Related Molecular Network; d Department of General Surgery, Division of Pancreatic Surgery, West China Hospital, Sichuan University; e Department of General Surgery, Division of Thyroid Surgery, West China Hospital, Sichuan University; f Department of Vascular Surgery, University Hospital of Chengdu University of Traditional Chinese Medicine, Chengdu, People’s Republic of China; g Department of Economics, Keio University, Minato city, Tokyo, Japan

**Keywords:** diabetes, epidemiology, pancreatectomy, risk factors

## Abstract

**Background and aims:**

Partial pancreatectomy, commonly used for chronic pancreatitis, or pancreatic lesions, has diverse impacts on endocrine and metabolism system. The study aims to determine the global prevalence of new-onset, worsening, and resolution of diabetes following partial pancreatectomy.

**Methods:**

The authors searched PubMed, Embase, Web of Science, and Cochrane Library from inception to October, 2023. DerSimonian-Laird random-effects model with Logit transformation was used. Sensitivity analysis, meta-regression, and subgroup analysis were employed to investigate determinants of the prevalence of new-onset diabetes.

**Results:**

A total of 82 studies involving 13 257 patients were included. The overall prevalence of new-onset diabetes after partial pancreatectomy was 17.1%. Univariate meta-regression indicated that study size was the cause of heterogeneity. Multivariable analysis suggested that income of country or area had the highest predictor importance (49.7%). For subgroup analysis, the prevalence of new-onset diabetes varied from 7.6% (France, 95% CI: 4.3–13.0) to 38.0% (UK, 95% CI: 28.2–48.8, *P*<0.01) across different countries. Patients with surgical indications for chronic pancreatitis exhibited a higher prevalence (30.7%, 95% CI: 21.8–41.3) than those with pancreatic lesions (16.4%, 95% CI: 14.3–18.7, *P*<0.01). The type of surgical procedure also influenced the prevalence, with distal pancreatectomy having the highest prevalence (23.7%, 95% CI: 22.2–25.3, *P*<0.01). Moreover, the prevalence of worsening and resolution of preoperative diabetes was 41.1 and 25.8%, respectively.

**Conclusions:**

Postoperative diabetes has a relatively high prevalence in patients undergoing partial pancreatectomy, which calls for attention and dedicated action from primary care physicians, specialists, and health policy makers alike.

## Introduction

Highlights

- Partial pancreatectomy carries the potential of triggering new-onset diabetes (referred to as type 3c diabetes).
- The global prevalence of new-onset diabetes after partial pancreatectomy was found to be 17.1% with significant heterogeneity influenced by macro factors such as regional variation, development, and income levels as well as micro factors including indication and procedure of surgery.
- Postoperative diabetes has a relatively high prevalence in patients undergoing partial pancreatectomy, which calls for attention and dedicated action.

Pancreatoduodenectomy (PD) and distal pancreatectomy (DP) are well-established treatment procedures used worldwide for chronic pancreatitis, benign or (potentially) malignant pancreatic lesions^1–3^. However, the implementation of PD and DP is associated with the loss of upper gastrointestinal and pancreato-biliary parenchyma, leading to impaired upper gastrointestinal functions^4,5^. To mitigate the substantial decrease in functional capacity of the upper gastrointestinal tract, some organ-preserving pancreatectomy procedures have been developed, including duodenum-preserving pancreatic head resection (DPPHR), pancreatic head resection with segmental duodenectomy (PHRSD), central pancreatectomy (CP), and tumor enucleation (TEU)^6–8^. These procedures aim to preserve pancreatic tissue, gastric antrum, duodenum, and common bile duct for maintaining the functionality of the residual pancreatic parenchyma and the upper gastrointestinal tract^9^.

It is widely acknowledged that partial pancreatectomy carries the potential of developing new-onset diabetes (referred to as type 3c diabetes) as well as exacerbating or resolving preexisting diabetes^10^. However, the real-world dynamics of diabetes development, progression, and changes following partial pancreatic resection still require further investigation. Certain macro factors that may contribute to the emergence of new-onset diabetes such as regional variation, socioeconomic class, and medical level have not been taken into account in previous studies^11,12^. Additionally, with regard to significant heterogeneity between different clinical studies, a comprehensive analysis of the preoperative, perioperative, and postoperative factors beyond indication and procedure of surgery is required to undertake. Moreover, the assessment of the worsening and resolution of diabetes following partial pancreatectomy has not been conducted in current systematic reviews^9,13^.

In this study, we aim to map the global new-onset, worsening, and resolution of diabetes following partial pancreatectomy. We performed a systematic review and meta-analysis through mining the existing data on postoperative diabetes after pancreatic resection. The study will broaden our knowledge of surgery-related postoperative changes of pancreatic endocrine function and provide evidence for clinical decision-making.

## Methods

This systematic review follows the recommendations of the (Assessing the methodological quality of systematic reviews) AMSTAR (Supplemental Digital Content 1, http://links.lww.com/JS9/B614) guidelines and is consistent with the preferred reporting items for systematic reviews and meta-analyses (PRISMA) statement (Supplemental Digital Content 2, http://links.lww.com/JS9/B615), (Supplemental Digital Content 3, http://links.lww.com/JS9/B616)^14,15^.

### Searching strategy and study selection

An extensive literature search was conducted using electronic database of PubMed, Embase, Web of Science, and the Cochrane Library. We searched articles with related terms ‘pancreatectomy’, ‘pancreatoduodenectomy’, ‘duodenum-preserving pancreatic head resection’, ‘pancreatic head resection with segmental duodenectomy’, ‘distal pancreatectomy’, ‘pancreatic left resection’, ‘spleno-pancreatectomy’, ‘central pancreatectomy’, ‘pancreatic middle segment resection’, ‘tumor enucleation’, ‘diabetes’, and ‘endocrine insufficiency’ (Supplementary Methods, Supplemental Digital Content 4, http://links.lww.com/JS9/B617).

We included studies for meta-analysis as follows: (1) a cohort study or case–control study fully published in English; (2) identified patients undergoing partial pancreatectomy; (3) reported number of preoperative and postoperative diabetes. We excluded studies for meta-analysis as follows: (1) individuals less than 18 years; (2) no sufficient information for data extraction.

### Selection criteria

The studies were meticulously evaluated following predetermined criteria. The preliminary screening of titles and abstracts was performed by two reviewers independently. To ensure accuracy, an additional investigator critically reviewed a randomly selected 10% subset of the studies. Subsequently, the full texts of potentially relevant articles were thoroughly examined by any two of the authors, and any disparities were resolved through group discussion or, if necessary, by a fifth reviewer. Consensus was successfully attained in all cases, establishing a high level of agreement among the reviewers.

### Data extraction

We extracted data at all levels reported in the study, including first author of the study, time of publication, study period, country or area, geographic region, income of country or area assessed by World Bank, the level of country development, study size, study population, indication of surgery, procedure and process of surgery, length of hospital stay, postoperative complications, duration of follow-up, diabetes diagnosis and the prevalence of new-onset, worsening and resolution of diabetes. Two authors independently reviewed and extracted data from the included studies by utilizing a custom-designed data extraction form tailored to the requirements of this investigation. Data were then cross-validated to guarantee accuracy by any of two authors. In cases where duplicate data were identified, the entry with the smaller sample size or shorter follow-up duration was excluded to prevent redundancy.

### Quality assessment and statistical analysis

The quality assessment of the 82 included studies was conducted using the Newcastle–Ottawa Scale (NOS) (Supplementary Table 1, Supplemental Digital Content 4, http://links.lww.com/JS9/B617). No studies were excluded based on their quality scores to ensure transparency and encompass all available evidence in this domain. Consistency checks were conducted and the Metaprop module within the R-4.2.2 statistical software package was employed for meta-analysis. A 95% CI was estimated using the Wilson score method, and the pooled prevalence was calculated using the DerSimonian-Laird random-effects model with Logit transformation. The heterogeneity among the included studies was evaluated through the Cochran Q statistics and *I* ^2^ statistics. Estimates with a *P*-value less than .05 for the Q-statistic and an *I* ^2^ value of 50% or higher were considered to indicate moderate heterogeneity. Given the anticipated heterogeneity in global data, a random-effects model was employed to pool the prevalence of new-onset, worsening and resolution of diabetes. Focusing on the significant heterogeneity associated with new-onset diabetes, we employed a meticulous sensitivity analysis approach by conducting a comprehensive series of leave-one-out diagnostic tests. Furthermore, the results were validated using a dedicated function within the metafor package to enhance the reliability and validity of our findings. As no outliers were identified through the sensitivity analysis, meta-regression was then conducted using a mixed-effects model. The considered covariates included country or area, geographic region, income of country or area, development level of country, study size, and study quality score. Subsequently, multivariable meta-regression (multimodel inference) was carried out using the ‘dmetar’ package to determine the best-fitting predictor combinations and identify the most significant overall predictors. Subgroup analyses were performed to assess potential confounding effects of heterogeneity. The difference between groups was assessed using a *P*-value, with a threshold of *P*<0.05 indicating a statistical significance.

## Results

### Literature search and study characteristics

**Figure 1:**
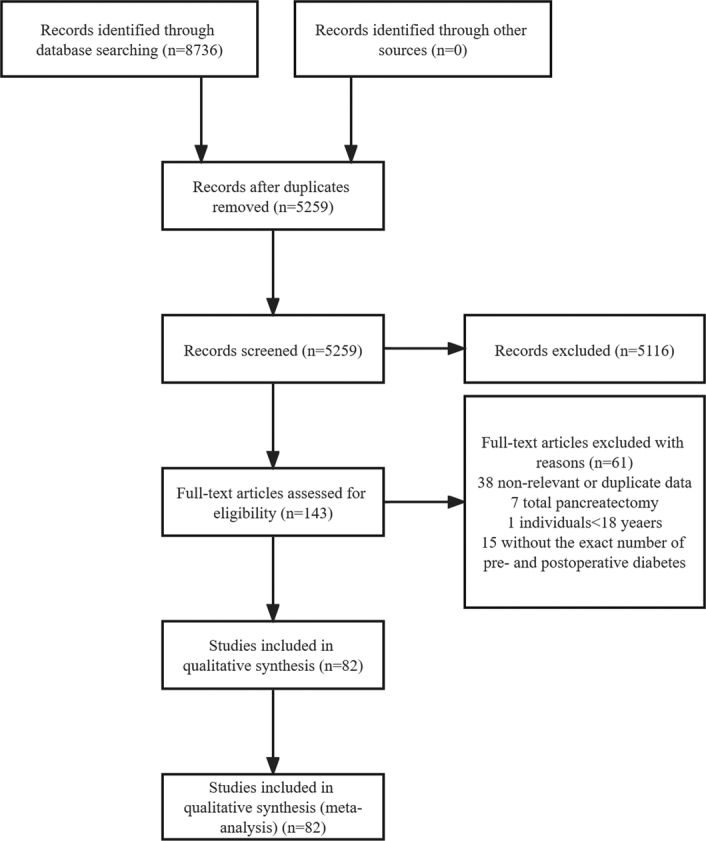
Study selection.

A total of 8736 records were identified. After eliminating duplicates, 5259 records remained. We screened the titles and abstracts and excluded 5116 ineligible records. The full texts of the remaining 143 records were evaluated for eligibility, with 61 being excluded. Ultimately, the analysis comprised 82 eligible studies involving 13 257 patients undergoing partial pancreatectomy, with 11 064 allocated to examine new-onset diabetes, 607 for worsening diabetes, and 1233 for resolution of diabetes (Fig. 1, Supplementary Table 1, Supplemental Digital Content 4, http://links.lww.com/JS9/B617)^16–97^. The quality assessment scores for the included studies were presented in Supplementary Table 1 (Supplemental Digital Content 4, http://links.lww.com/JS9/B617).

### Global new-onset, worsening, and resolution of diabetes prevalence in patients following partial Pancreatectomy

**Figure 2:**
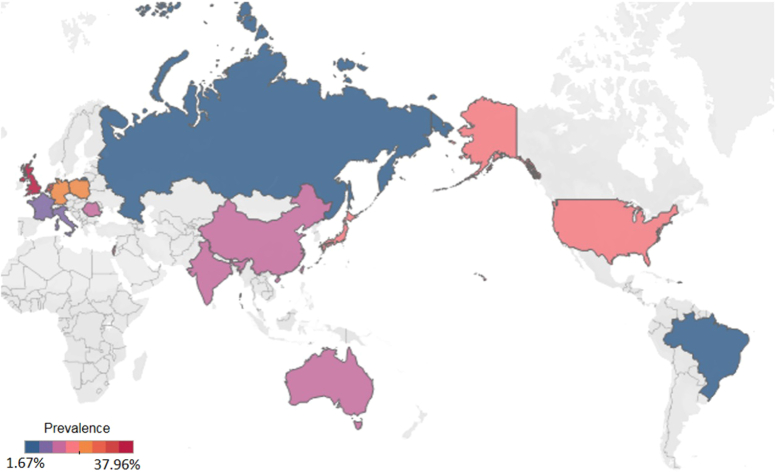
Global prevalence of new-onset diabetes following partial pancreatectomy.

We identified 1827 patients developing new-onset diabetes after partial pancreatectomy with an overall pooled prevalence of 17.1% (95% CI: 15.0–19.5, *I* ^2^=84.0%, Table 1, Fig. 2).

**Table 1:** Subgroup analysis for new-onset diabetes following partial pancreatectomy.

	Studies	New-onset diabetes	Patients	Prevalence (95% CI)	*P*	*I* ^2^ (%)
Overall population	82	1827	11064	17.1 (15.0–19.5)	–	84.0
By geographic regions					0.1	
Eastern Asia	31	1211	7392	15.3 (12.9–18.0)		75.8
Northern America	19	260	1782	17.7 (12.7–24.1)		84.6
Central Europe	11	183	807	23.4 (14.7–35.0)		88.9
Western Europe	10	124	592	19.6 (10.4–33.7)		88.5
Southern Europe	6	32	292	11.6 (7.1–18.2)		45.1
Oceania	1	11	95	11.6 (6.5–19.7)		–
Eastern Europe	1	2	49	4.1 (1.0–14.9)		–
Southern America	1	0	29	1.7 (0.1–21.7)		–
Southern Asia	1	2	14	14.3 (3.6–42.7)		–
Western Asia	1	2	12	16.7 (4.2–47.7)		–
By country or area					<0.01	
USA	19	260	1782	17.7 (12.7–24.1)		84.6
China	11	1007	6075	12.0 (9.1–15.8)		84.2
Korea	10	139	894	17.3 (12.5–23.4)		72.6
Germany	10	180	793	23.3 (14.2–35.7)		90.0
Japan	10	65	423	16.6 (10.7–24.8)		66.1
France	5	9	188	7.6 (4.3–13.0)		0.0
Italy	4	18	188	10.6 (5.3–20.1)		50.7
UK	3	67	177	38.0 (28.2–48.8)		49.4
Netherlands	2	48	227	32.3 (4.7–82.0)		97.1
Romania	2	14	104	12.7 (5.2–28.0)		55.8
Australia	1	11	95	11.6 (6.5–19.7)		–
Russia	1	2	49	4.1 (1.0–14.9)		–
Brazil	1	0	29	1.7 (0.1–21.7)		–
Poland	1	3	14	21.4 (7.1–49.4)		–
India	1	2	14	14.3 (3.6–42.7)		–
Israel	1	2	12	16.8 (4.2–47.7)		–
By income					0.01	
High	67	804	4842	18.0 (15.0–21.4)		83.8
Upper-middle	15	1023	6222	12.2 (9.5–15.6)		79.6
By development					0.03	
Developed	67	801	4857	17.2 (14.2–20.6)		84.0
Developing	15	1026	6207	12.4 (9.6–15.7)		79.5
By study size					0.04	
<100	63	491	2618	18.1 (15.0–21.8)		75.8
≥100	19	1336	8446	13.6 (11.0–16.7)		90.9
By study period					0.2	
Before 2010	53	1486	8689	18.7 (15.7–22.0)		86.1
After 2010	7	53	339	14.6 (7.7–25.9)		76.1
By study quality score					0.5	
<7	16	129	922	17.5 (15.2–20.1)		83.1
≥7	66	1698	10142	14.4 (8.5–23.4)		86.5
By duration of follow-up					0.06	
<6 months	11	50	842	9.5 (6.0–14.8)		50.0
≥6 months	25	186	1279	15.6 (11.7–20.6)		71.9

**Figure 3:**
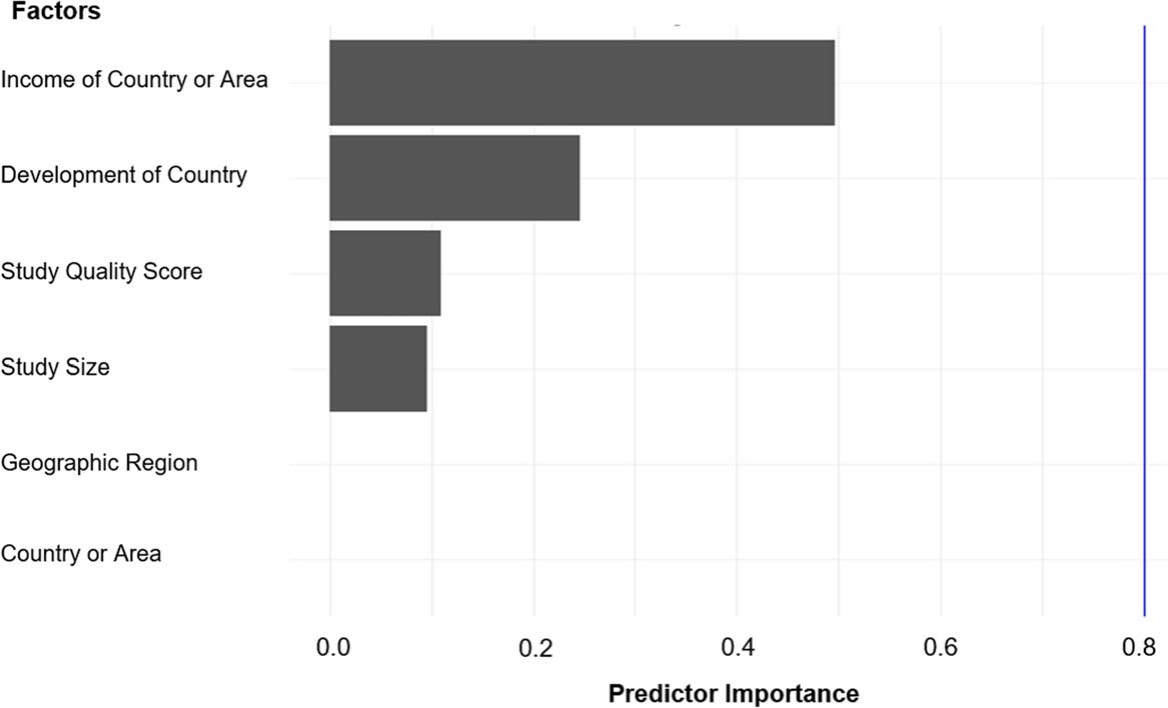
Results of multivariable meta-regression analysis.

To gain a deeper understanding of the heterogeneity, we conducted a sensitivity analysis by performing a set of leave-one-out diagnostic tests (Supplementary Table 2, Supplemental Digital Content 4, http://links.lww.com/JS9/B617) and the results were further verified using a build-in function in metafor (Supplementary Figure 1, Supplemental Digital Content 4, http://links.lww.com/JS9/B617). Regrettably, neither approach identified the outliers. To further explore the source of heterogeneity, meta-regression analysis was performed. Our univariate meta-regression model indicated that country or area (R^2^=0, *P*=0.8), geographic region (R^2^=0, *P*=0.9), income of country or area (R^2^=0, *P*=0.9), development level of country (R^2^=0, *P*=0.9), study quality score (R^2^=0, *P*=0.9) were not significantly associated with heterogeneity (Supplementary Table 3, Supplemental Digital Content 4, http://links.lww.com/JS9/B617). The source of heterogeneity across the studies, identified by univariate meta-regression analyses, was study size (R^2^=0.6, *P*<0.01, Supplementary Table 3, Supplemental Digital Content 4, http://links.lww.com/JS9/B617). By performing multivariable meta-regression, we found that income of country or area exhibited the highest predictor importance of 49.7% (Fig. 3).

Subgroup analysis was conducted to validate the findings obtained from the meta-regression analysis. Regarding the geographic region with a minimum of five studies, the prevalence of new-onset diabetes ranged from 11.6% (Southern Europe, 95% CI: 7.1–18.2) to 23.4% (Central Europe, 95% CI: 14.7–35.0, *P*=0.1, Table 1). Among countries with at least three studies, UK exhibited the highest prevalence (38.0%, 95% CI: 28.2–48.8), followed by Germany (23.3%, 95% CI: 14.2–35.7), USA (17.7%, 95% CI: 12.7–24.1), Korea (17.3%, 95% CI: 12.5–23.4), Japan (16.6%, 95% CI: 10.7–24.8), China (12.0%, 95% CI: 9.1–15.8), Italy (10.6%, 95% CI: 5.3–20.1), and France (7.6%, 95% CI: 4.3–13.0, *P*<0.01, Table 1). When considering the income level, countries or areas with high-income displayed a greater prevalence of 18.0% (95% CI: 15.0–21.4) compared to those with upper-middle income (12.2%, 95% CI: 9.5–15.6, *P*=0.01, Table 1). Besides, developed countries (17.2%, 95% CI: 14.2–20.6) shared a higher postoperative diabetes prevalence than that of developing countries (12.4%, 95% CI: 9.6–15.7, *P*=0.03, Table 1). Studies included with fewer than 100 patients (18.1%, 95% CI: 15.0–21.8) exhibited significantly higher prevalence of diabetes than those with more than 100 patients (13.6%, 95% CI: 11.0–16.7, *P*=0.04, Table 1). Considering the study period, studies conducted before 2010 (18.7%, 95% CI: 15.7–22.0) had a slightly higher prevalence of diabetes than those performed after 2010 (14.6%, 95% CI: 7.7–25.9, *P*=0.2, Table 1). The quality assessment revealed 46 studies with a rating below 7 points (17.5%, 95% CI: 15.2–20.1), and 36 studies with a rating above 7 points (14.4%, 95% CI: 8.5–23.4, *P*=0.5, Table 1). In terms of the duration of follow-up, 11 studies reported less than 6 months (9.5%, 95% CI: 6.0–14.8), while 25 studies reported more than 6 months (15.6%, 95% CI: 11.7–20.6, *P*=0.06, Table 1).

Additionally, we assessed the preoperative, perioperative, and postoperative risk factors contributing to the development of new-onset diabetes. It was observed that patients with surgical indications related to chronic pancreatitis exhibited a higher prevalence (30.7%, 95% CI: 21.8–41.3) compared to patients with surgical indications of benign or (potentially) malignant pancreatic lesions (16.4%, 95% CI: 14.3–18.7, *P*<0.01, Table 2). Concerning the type of surgery, patients undergoing DP (23.7%, 95% CI: 22.2–25.3) had the highest prevalence, followed by DPPHR (22.8%, 95% CI: 12.8–37.4), PD (15.8%, 95% CI: 14.9–16.8), PHRSD (10.6%, 95% CI: 4.0–25.3), CP (9.4%, 95% CI: 7.3–12.0) and TEU (6.9%, 95% CI: 2.9–15.6, *P*<0.01, Table 2, Supplementary Table 4, Supplemental Digital Content 4, http://links.lww.com/JS9/B617). However, minimal differences were observed among age (≤ 49/50–64 /≥65, 17.8% vs. 20.1% vs. 20.9%, *P*=0.5), sex (male/female, 20.4 vs. 17.6%, *P*=0.4), BMI (< 25 /≥25, 20.7 vs. 24.5%, *P*=0.7), hypertension (Yes /No, 18.0 vs. 14.3%, *P*=0.3), dyslipidemia (Yes /No, 21.8 vs. 15.1%, *P*=0.4), peptic ulcer (Yes / No, 19.7 vs. 17.9%, *P*=0.7), kidney disease (Yes /No, 25.5 vs. 13.8%, *P*=0.4), blood glucose levels (euglycemia / impaired fasting glucose or impaired glucose tolerance, 9.9 vs. 18.3%, *P*=0.4), type of procedure (open/laparoscopic/robotic, 18.1% vs. 17.4% vs. 7.1%, *P*=0.08), operation time (< 250 min/≥ 250 min, 20.1 vs. 10.8%, *P*=0.2), blood loss (< 100 ml /≥ 100 ml, 7.3 vs. 15.7%, *P*=0.2), blood transfusion (Yes / No, 16.7 vs. 24.0%, *P*=0.3) and postoperative complications (Yes / No, 16.4 vs. 14.8%, *P*=0.7, Table 2). We also conducted an evaluation of insulin utilization in patients with new-onset diabetes. The findings revealed that an impressive 52.9% (95% CI: 41.9–63.6) of patients relied on insulin therapy subsequent to the onset of diabetes (Supplementary Figure 2, Supplemental Digital Content 4, http://links.lww.com/JS9/B617).

**Table 2:** Pooled estimates of risk factors for new-onset diabetes following partial pancreatectomy.

	Studies	New-onset diabetes	Patients	Prevalence (95% CI)	*P*	*I* ^2^ (%)
Preoperative risk factors						
Age					0.5	
≤49	9	257	1463	17.8 (15.3–20.6)		10.5
50–64	9	347	1828	20.1 (16.4–24.4)		26.3
≥65	8	348	2122	20.9 (14.7–28.8)		62.8
Sex					0.4	
Male	18	649	3556	20.4 (16.0–25.5)		69.2
Female	18	437	2707	17.6 (13.9–22.0)		58.1
BMI					0.7	
BMI<25	3	27	134	20.7 (14.6–28.5)		0.0
BMI≥25	3	11	50	24.5 (10.4–47.5)		52.3
Hypertension					0.3	
Yes	4	376	2050	18.0 (12.7–24.9)		81.1
No	4	594	3609	14.3 (10.0–20.1)		90.4
Dyslipidemia					0.4	
Yes	3	105	499	21.8 (9.3–43.0)		90.0
No	3	865	5148	15.1 (11.0–20.4)		93.8
Peptic ulcer					0.7	
Yes	2	529	2943	19.7 (12.8–29.2)		96.1
No	2	415	2381	17.9 (14.9–21.4)		73.6
Kidney disease					0.4	
Yes	2	11	38	25.5 (6.4–62.9)		39.2
No	2	327	1695	13.8 (5.0–32.9)		96.5
Blood glucose level					0.4	
Euglycemia	6	32	528	9.9 (2.9–28.9)		88.2
IFG/IGT	6	75	512	18.3 (8.2–36.1)		89.3
Indication of surgery					<0.01	
Chronic pancreatitis	17	240	832	30.7 (21.8–41.3)		83.4
Benign or (potentially) malignant lesions	42	1027	6602	16.4 (14.3–18.7)		58.4
Perioperative risk factors						
Type of surgery					<0.01	
PD	36	939	6392	15.8 (14.9–16.8)		80.2
DPPHR	11	93	389	22.8 (12.8–37.4)		82.7
PHRSD	3	9	98	10.6 (4.0–25.3)		49.5
CP	31	50	781	9.4 (7.3–12.0)		10.1
DP	37	715	3162	23.7 (22.2–25.3)		72.5
TEU	2	4	73	6.9 (2.9–15.6)		0.0
Type of procedure					0.08	
Open	6	6	61	18.1 (4.0–53.7)		61.3
Laparoscopic	11	49	314	17.4 (12.9–23.1)		10.4
Robotic	4	4	82	7.1 (3.2–14.9)		0.0
Operation time					0.2	
<250 min	7	6	49	20.1 (10.9–34.2)		0.0
≥250 min	6	2	47	10.8 (4.5–23.7)		0.0
Blood loss					0.2	
<100 ml	6	0	51	7.3 (2.3–20.7)		0.0
≥100 ml	7	14	120	15.7 (10.0–23.7)		0.0
Blood transfusion					0.3	
Yes	2	12	76	16.7 (10.0–26.7)		0.0
No	2	21	88	24.0 (16.2–34.0)		0.0
Postoperative risk factors						
Postoperative complications					0.7	
Yes	9	30	239	16.4 (9.1–27.6)		47.9
No	9	36	320	14.8 (9.0–23.2)		40.8

CP, central pancreatectomy; DP, distal pancreatectomy; DPPHR, duodenum-preserving pancreatic head resection; IFG, impaired fasting glucose; IGT, impaired glucose tolerance; PD, pancreatoduodenectomy; PHRSD, pancreatic head resection with segmental duodenectomy; TEU, tumor enucleation.

Moreover, we estimated the pooled prevalence of worsening and resolution of diabetes in patients with preoperative diabetes. The results indicated that 277 patients (41.1%, 95% CI: 31.4–51.6) with preoperative diabetes experienced a deterioration in metabolic control and 255 patients (25.8%, 95% CI: 19.3–33.7) recovered from previously diagnosed diabetes (Supplementary Figure 3, Supplemental Digital Content 4, http://links.lww.com/JS9/B617).

## Discussion

Partial pancreatectomy for chronic pancreatitis, benign or (potentially) malignant pancreatic lesions is associated with the development, exacerbation, and resolution of diabetes. The global prevalence of new-onset diabetes was found to be 17.1% with significant difference between different countries. Univariate meta-regression indicated that study size was the causes of heterogeneity and multivariable analysis suggested that income of country or area has the highest predictor importance. Furthermore, a notable disparity of postoperative diabetes prevalence was identified with statistical significance due to the indication and type of surgery. Once developing diabetes, more than 50% of individuals required insulin therapy to effectively manage their blood glucose levels. It is noteworthy that pancreatic resection not only carried the potential to trigger new-onset or worsening diabetes but also held promise for ameliorating preexisting diabetes.

Recent systematic reviews have aimed to estimate the prevalence of new-onset diabetes following partial pancreatectomy^9,11–13^. However, to our knowledge, this study represents the most comprehensive review to date, incorporating the largest number of studies on postoperative diabetes worldwide. Our investigation involved a series of rigorous and extensive analysis, first considering certain macro-level factors such as country and regional disparities, as well as economic development level, to examine their potential impact on the occurrence of postoperative diabetes. In addition, we conducted a comprehensive examination of preoperative, perioperative, and postoperative variables, while also scrutinizing the global prevalence of worsening and resolution of diabetes following partial pancreatectomy. Our study has several limitations. First, despite its comprehensive nature, there is a notable scarcity of research studies from developing countries with lower-middle or low-income. This lack of representation hampers the generalizability of our findings. Second, the metabolic information in some studies were self-reported, and the diagnosis of diabetes did not strictly adhere to WHO-criteria. This potential reporting bias may introduce uncertainties and affect the accuracy of the results. Third, it is reported that the proportion of pancreas removal was associated with postoperative diabetes^38,67^. However, due to the limited availability of studies, we failed to further analyze these data. Lastly, because there was insufficient data from the included studies, our meta-analysis did not thoroughly examine the impact of preoperative diabetes types, surgical indications, and specific surgical procedures on the exacerbation and resolution of diabetes.

Our findings indicated that some western countries, including UK, Germany, and USA, exhibited higher prevalence of postoperative diabetes compared to some eastern countries, such as China, Korea, and Japan. This observed disparity can be attributed to variations in dietary habits in different countries. Western countries often embrace a diet characterized by a greater consumption of processed foods, which are typically abundant in unhealthy fats, added sugars, and refined carbohydrates. These dietary patterns may lead to an increased risk of developing diabetes^98,99^. In contrast, eastern countries, particularly China, Korea, and Japan, traditionally prioritize diets rich in whole grains, vegetables, and seafood, which are generally regarded as healthier choices. These dietary preferences may contribute to a lower prevalence of postoperative diabetes in these regions^100,101^. Notably, countries such as Italy and France, renowned for their adherence to internationally acclaimed health-conscious dietary pattern known as the Mediterranean diet, showed the lowest prevalence of postoperative diabetes^102^.

Furthermore, our analysis revealed that higher prevalence of new-onset diabetes in developed and high-income countries compared to developing countries with upper-middle income. Indeed, it is acknowledged that significant strides have been achieved in healthcare infrastructure in developing countries with an upper-middle income level over the past decade^103^. In the realm of pancreatectomy, these countries have made noteworthy advancements in critical aspects, encompassing preoperative assessments, surgical techniques, and postoperative management, thereby bringing them into greater conformity with global standards^104,105^. It is interesting that a considerable proportion of patients undergoing pancreatic resection in these countries often engage in physically demanding occupations due to their relatively underdeveloped economies. This specific employment setting could potentially operate as a mitigating factor, reducing the prevalence of postoperative diabetes^106^. Although the lack of relevant data and the restricted rigor of research design prevented us from including data from developing with lower-middle or low incomes in our meta-analysis, we are able to present a general picture of the state of pancreatectomy in these areas. In comparison to high-income countries that possess cutting-edge medical infrastructure, such as state-of-the-art operating rooms, CT scanners, MRI machines, and robotic surgical systems, lower-middle and low-income countries often grapple with substantial challenges related to basic healthcare infrastructure^107^. In addition, high-income countries benefit from a well-established cadre of highly proficient surgeons who specialize in the complexities of pancreatic surgery^107^. Conversely, lower-middle and low-income countries frequently encounter the formidable obstacle of inadequate specialized training programs and a shortage of experienced surgeons possessing the necessary expertise to undertake intricate pancreatic resections^107^. The insufficiency of healthcare infrastructure and the scarcity of specialized medical professionals have markedly increased the incidence of postoperative complications among patients undergoing pancreatic resection in these nations^108,109^. It is possible that these factors have contributed to a dearth of research on the long-term outcomes of pancreatic resections within these nations, such as exocrine and endocrine insufficiency.

A potential explanation for the elevated occurrence of new-onset diabetes in patients undergoing partial pancreatectomy for chronic pancreatitis is likely attributed to the progressive destruction of pancreatic parenchyma^110^. Conversely, in patients with benign or (potentially) malignant pancreatic lesions, the remaining pancreatic parenchyma is relatively healthy^13^. Notably, following tumor resection, the impaired pancreatic β-cell function caused by tumor cells shows a remarkable improvement, as well as the degree of peripheral insulin resistance^111–114^. Consequently, these patients have a lower risk of developing new-onset diabetes. In terms of different surgical procedures, it is widely acknowledged that the extent and location of pancreas removal are significantly associated with the development of diabetes^10^. Our study, based on empirical data, demonstrated that specific pancreas-preserving techniques, such as CP and TEU, effectively reduced the risk of postoperative diabetes. Previous research has indicated that islet density and distribution are approximately two times higher in the tail region compared to that in the head and body regions, which may account for the highest risk of new-onset diabetes in patients undergoing the DP procedure^115^. The impact of duodenal preservation on the emergence of postoperative diabetes remains a matter of controversy. The resection of the duodenum may induce alterations in incretin secretion, including a decrease in gastric inhibitory polypeptide secretion and an increase in glucagon-like peptide 1 (GLP-1) secretion, which regulate β-cell function and insulin sensitivity in peripheral tissues^10,116,117^.

Another interesting finding was that there was no statistically significant disparity in postoperative diabetes prevalence between two BMI groups (BMI <25 and BMI ≥25). Although previous studies have suggested that overweight and obese individuals may exhibit a relatively compromised pancreatic parenchyma, commonly referred to as soft pancreatic tissue, we posit that the impairment in pancreatic β-cell function resulting from fatty infiltration is much less severe compared to the pancreatic diseases that necessitate surgical intervention^118^. In fact, it appears that the degree of damage to the pancreatic parenchyma is nearly indistinguishable between both groups. Within these two groups, we still believed that the primary determinant for postoperative diabetes may be the insufficient insulin secretion resulting from the resection of pancreatic parenchyma. Furthermore, the relatively limited number of studies included in the subgroup analysis may contribute to the absence of statistically significant difference. Additional research efforts may be merited to conduct a more comprehensive exploration of this matter.

The concern is not groundless. First, diabetes exerts a profound impact on one’s quality of life. Belyaev *et al*.^42^ provided evidence of patients enduring postoperative endocrine or exocrine insufficiency, or both, reporting significant declines in their physical well-being. In a comparative study examining the quality of life after partial pancreatic resection, postoperative diabetes exhibited the most detrimental effects on leisure activities and physical functioning^85^. Second, a remarkable 52.9% of patients developing new-onset diabetes required insulin therapy to effectively manage their blood glucose levels, and 41.1% of individuals with preoperative diabetes experienced a deterioration in metabolic control. Furthermore, despite certain similarities between type 3c diabetes and type 2 diabetes, type 3c diabetes presents greater challenges in blood glucose management due to notable fluctuations associated with the deficiency of pancreatic polypeptide and exogenous insulin treatment^119,120^. However, it is important to recognize that pancreatic resection does not solely yield negative outcomes. Notably, 25.8% of patients experienced a restoration from previously diagnosed diabetes, although the underlying mechanism needs further investigation.

In conclusion, postoperative diabetes has a relatively high prevalence in patients undergoing partial pancreatectomy and poses negative effects on their life. This calls for attention and dedicated action from primary care physicians, specialists, and health policy makers alike.

## Ethical approval

Our research does not require ethical review.

## Consent

Not applicable.

## Sources of funding

This work is supported by the National Natural Science Foundation of China (82070846), EFSD and Lilly EXPLORING AND APPLYING NEW STRATEGIES IN DIABETES (EXPAND) Program, Sichuan Science and Technology Program (2021ZYCD016, 2022YFS0308, 2023YFS0123), and Chengdu Science and Technology Program (2023-GH02-00083-HZ).

## Author contribution

J.W., J.L., and X.Z.: designed the study; J.W., Y.O., J.C., and Z.Y.: carried out the research; J.W., Y.O., J.L., and X.Z. wrote the manuscript. Z.W., K.W., D.Y., Y.G., and Y.L.: critically revised the manuscript. All authors contributed and approved the final version of the manuscript.

## Conflicts of interest disclosure

The authors declare no conflicts of interest.

## Research registration unique identifying number (UIN)

1. Name of the registry: PROSEPRO.
2. Unique identifying number or registration ID: CRD42023457885.
3. Hyperlink to your specific registration (must be publicly accessible and will be checked): https://www.crd.york.ac.uk/prospero/display_record.php?ID=CRD42023457885

## Guarantor

The Guarantor is Prof Xiaofeng Zheng.

## Data availability statement

Datasets generated during the current study are available upon reasonable request.

## Provenance and peer review

Not commissioned, externally peer-reviewed.

## Supplementary Material

SUPPLEMENTARY MATERIAL

## References

[1] Büchler MW Wagner M Schmied BM . Changes in morbidity after pancreatic resection: toward the end of completion pancreatectomy. Arch Surg 2003;138:1310–1314. 14662530 10.1001/archsurg.138.12.1310

[2] Cameron JL He J . Two thousand consecutive pancreaticoduodenectomies. J Am Coll Surg 2015;220:530–536. 25724606 10.1016/j.jamcollsurg.2014.12.031

[3] Trede M Schwall G Saeger HD . Survival after pancreatoduodenectomy. 118 consecutive resections without an operative mortality. Ann Surg 1990;211:447–458. 2322039 10.1097/00000658-199004000-00011 PMC1358031

[4] Beger HG Poch B Mayer B . New onset of diabetes and pancreatic exocrine insufficiency after pancreaticoduodenectomy for benign and malignant tumors: a systematic review and meta-analysis of long-term results. Ann Surg 2018;267:259–270. 28834847 10.1097/SLA.0000000000002422

[5] Borja-Cacho D Al-Refaie WB Vickers SM . Laparoscopic distal pancreatectomy. J Am Coll Surg 2009;209:758–765. 19959046 10.1016/j.jamcollsurg.2009.08.021

[6] Beger HG Mayer B Rau BM . Parenchyma-sparing, limited pancreatic head resection for benign tumors and low-risk periampullary cancer–a systematic review. J Gastrointest Surg 2016;20:206–217. 26525207 10.1007/s11605-015-2981-2

[7] Iacono C Verlato G Ruzzenente A . Systematic review of central pancreatectomy and meta-analysis of central versus distal pancreatectomy. Br J Surg 2013;100:873–885. 23640664 10.1002/bjs.9136

[8] Zhou Y Zhao M Wu L . Short- and long-term outcomes after enucleation of pancreatic tumors: an evidence-based assessment. Pancreatology 2016;16:1092–1098. 27423534 10.1016/j.pan.2016.07.006

[9] Beger HG Mayer B Vasilescu C . Long-term metabolic morbidity and steatohepatosis following standard pancreatic resections and parenchyma-sparing, local extirpations for benign tumor: a systematic review and meta-analysis. Ann Surg 2022;275:54–66. 33630451 10.1097/SLA.0000000000004757

[10] Mezza T Cefalo CMA Cinti F . Endocrine and metabolic insights from pancreatic surgery. Trends Endocrinol Metab 2020;31:760–772. 32830029 10.1016/j.tem.2020.07.003

[11] Farrarons SS van Bodegraven EA Sauvanet A . Minimally invasive versus open central pancreatectomy: systematic review and meta-analysis. Surgery 2022;172:1490–1501. 35987787 10.1016/j.surg.2022.06.024

[12] Wu L Nahm CB Jamieson NB . Risk factors for development of diabetes mellitus (Type 3c) after partial pancreatectomy: a systematic review. Clin Endocrinol (Oxf) 2020;92:396–406. 32017157 10.1111/cen.14168

[13] De Bruijn KM van Eijck CH . New-onset diabetes after distal pancreatectomy: a systematic review. Ann Surg 2015;261:854–861. 24983994 10.1097/SLA.0000000000000819

[14] Shea BJ Reeves BC Wells G . AMSTAR 2: a critical appraisal tool for systematic reviews that include randomised or non-randomised studies of healthcare interventions, or both. BMJ (Clinical research ed) 2017;358:j4008. 10.1136/bmj.j4008 PMC5833365 28935701

[15] Page MJ McKenzie JE Bossuyt PM . The PRISMA 2020 statement: an updated guideline for reporting systematic reviews. Int J Surg 2021;88:105906. 33789826 10.1016/j.ijsu.2021.105906

[16] DiNorcia J Ahmed L Lee MK . Better preservation of endocrine function after central versus distal pancreatectomy for mid-gland lesions. Surgery 2010;148:1247–1254. 21134558 10.1016/j.surg.2010.09.003

[17] Hirono S Tani M Kawai M . A central pancreatectomy for benign or low-grade malignant neoplasms. J Gastrointest Surg 2009;13:1659–1665. 19488821 10.1007/s11605-009-0934-3

[18] Paiella S De Pastena M Faustini F . Central pancreatectomy for benign or low-grade malignant pancreatic lesions - a single-center retrospective analysis of 116 cases. Eur J Surg Oncol 2019;45:788–792. 30527222 10.1016/j.ejso.2018.11.021

[19] Brown KM Shoup M Abodeely A . Central pancreatectomy for benign pancreatic lesions. HPB 2006;8:142–147. 18333263 10.1080/13651820510037611 PMC2131409

[20] Lee DH Han Y Byun Y . Central pancreatectomy versus distal pancreatectomy and pancreaticoduodenectomy for benign and low-grade malignant neoplasms: a retrospective and propensity score-matched study with long-term functional outcomes and pancreas volumetry. Ann Surg Oncol 2020;27:1215–1224. 31898101 10.1245/s10434-019-08095-z

[21] Dumitrascu T Scarlat A Ionescu M . Central pancreatectomy versus spleen-preserving distal pancreatectomy: a comparative analysis of early and late postoperative outcomes. Dig Surg 2012;29:400–407. 23128466 10.1159/000343927

[22] Adham M Giunippero A Hervieu V . Central pancreatectomy: single-center experience of 50 cases. Arch Surg 2008;143:175–180; discussion 80-1. 18283143 10.1001/archsurg.2007.52

[23] Wu JM Ho TW Yang CY . Changes in glucose metabolism after distal pancreatectomy: a nationwide database study. Oncotarget 2018;9:11100–11108. 29541399 10.18632/oncotarget.24325 PMC5834261

[24] Litwin J Dobrowolski S Orłowska-Kunikowska E . Changes in glucose metabolism after Kausch-Whipple pancreatectomy in pancreatic cancer and chronic pancreatitis patients. Pancreas 2008;36:26–30. 18192877 10.1097/mpa.0b013e318137aa61

[25] Sato N Yamaguchi K Yokohata K . Changes in pancreatic function after pancreatoduodenectomy. Am J Surg 1998;176:59–61. 9683135 10.1016/s0002-9610(98)00105-6

[26] Lee SE Jang JY Hwang DW . Clinical efficacy of organ-preserving pancreatectomy for benign or low-grade malignant potential lesion. J Korean Med Sci 2010;25:97–103. 20052354 10.3346/jkms.2010.25.1.97 PMC2800014

[27] Dumitrascu T Dima S Stroescu C . Clinical value of spleen-preserving distal pancreatectomy: a case-matched analysis with a special emphasis on the postoperative systemic inflammatory response. J Hepato-biliary-Pancreat Sci 2014;21:654–662. 10.1002/jhbp.110 24799122

[28] Ocuin LM Sarmiento JM Staley CA . Comparison of central and extended left pancreatectomy for lesions of the pancreatic neck. Ann Surg Oncol 2008;15:2096–2103. 18521682 10.1245/s10434-008-9987-x

[29] Zhang RC Zhang B Mou YP . Comparison of clinical outcomes and quality of life between laparoscopic and open central pancreatectomy with pancreaticojejunostomy. Surg Endosc 2017;31:4756–4763. 28424909 10.1007/s00464-017-5552-7

[30] Li Y Wu W Zhang T . Comparison of long-term benefits of organ-preserving pancreatectomy techniques for benign or low-grade malignant tumors at the pancreatic head. Medicine 2017;96:e9420. 29390567 10.1097/MD.0000000000009420 PMC5758269

[31] Fujii T Kanda M Kodera Y . Comparison of pancreatic head resection with segmental duodenectomy and pylorus-preserving pancreatoduodenectomy for benign and low-grade malignant neoplasms of the pancreatic head. Pancreas 2011;40:1258–1263. 21705943 10.1097/MPA.0b013e318220b1c0

[32] Jang JY Kim SW Park SJ . Comparison of the functional outcome after pylorus-preserving pancreatoduodenectomy: pancreatogastrostomy and pancreatojejunostomy. World J Surg 2002;26:366–371. 11865376 10.1007/s00268-001-0234-x

[33] Sakata N Egawa S Rikiyama T . Computed tomography reflected endocrine function of the pancreas. J Gastrointest Surg 2011;15:525–532. 21181561 10.1007/s11605-010-1406-5

[34] Tariq M Jajja MR Maxwell DW . Diabetes development after distal pancreatectomy: results of a 10 year series. HPB 2020;22:1034–1041. 31718897 10.1016/j.hpb.2019.10.2440

[35] Lee CYC Depczynski B Poynten A . Diabetes-related outcomes after pancreatic surgery. ANZ J Surg 2020;90:2004–2010. 32691521 10.1111/ans.16129

[36] Mori Y Ohtsuka T Tsutsumi K . Different incretin responses after pancreatoduodenectomy and distal pancreatectomy. Pancreas 2012;41:455–460. 22422137 10.1097/MPA.0b013e3182319d7c

[37] Siegel JB Mukherjee R Lancaster WP . Distal pancreatectomy for pancreatitis in the modern era. J Surg Res 2022;275:29–34. 35219248 10.1016/j.jss.2022.01.016

[38] King J Kazanjian K Matsumoto J . Distal pancreatectomy: incidence of postoperative diabetes. J Gastrointest Surg 2008;12:1548–1553. 18543045 10.1007/s11605-008-0560-5

[39] Yun SP Seo HI Kim S . Does the pancreatic volume reduction rate using serial computed tomographic volumetry predict new onset diabetes after pancreaticoduodenectomy? Medicine 2017;96:e6491. 28353594 10.1097/MD.0000000000006491 PMC5380278

[40] Izbicki JR Bloechle C Knoefel WT . Duodenum-preserving resection of the head of the pancreas in chronic pancreatitis. A prospective, randomized trial. Ann Surg 1995;221:350–358. 7726670 10.1097/00000658-199504000-00004 PMC1234583

[41] Takada T Yasuda H Nagashima I . Duodenum-preserving total pancreatic head resection and pancreatic head resection with segmental duodenostomy. Nihon Geka Gakkai zasshi 2003;104:476–480. 12854495

[42] Belyaev O Herzog T Chromik AM . Early and late postoperative changes in the quality of life after pancreatic surgery. Langenbeck’s Arch Surg 2013;398:547–555. 23503698 10.1007/s00423-013-1076-3

[43] Wang ZZ Zhao GD Zhao ZM . An end-to-end pancreatic anastomosis in robotic central pancreatectomy. World J Surg Oncol 2019;17:67. 30981283 10.1186/s12957-019-1609-5 PMC6462313

[44] Cataldegirmen G Schneider CG Bogoevski D . Extended central pancreatic resection as an alternative for extended left or extended right resection for appropriate pancreatic neoplasms. Surgery 2010;147:331–338. 20004436 10.1016/j.surg.2009.10.027

[45] Orfanidis NT Loren DE Santos C . Extended follow-up and outcomes of patients undergoing pancreaticoduodenectomy for nonmalignant disease. J Gastrointest Surg 2012;16:80–87; discussion 7-8. 22058043 10.1007/s11605-011-1751-z

[46] van der Gaag NA van Gulik TM Busch OR . Functional and medical outcomes after tailored surgery for pain due to chronic pancreatitis. Ann Surg 2012;255:763–770. 22418009 10.1097/SLA.0b013e31824b7697

[47] Lemaire E O’Toole D Sauvanet A . Functional and morphological changes in the pancreatic remnant following pancreaticoduodenectomy with pancreaticogastric anastomosis. Br J Surg 2000;87:434–438. 10759738 10.1046/j.1365-2168.2000.01388.x

[48] Wu JM Ho TW Kuo TC . Glycemic change after pancreaticoduodenectomy: a population-based study. Medicine 2015;94:e1109. 26166104 10.1097/MD.0000000000001109 PMC4504605

[49] Hamilton L Jeyarajah DR . Hemoglobin A1c can be helpful in predicting progression to diabetes after Whipple procedure. HPB 2007;9:26–28. 18333109 10.1080/13651820600917286 PMC2020780

[50] Ferrara MJ Lohse C Kudva YC . Immediate post-resection diabetes mellitus after pancreaticoduodenectomy: incidence and risk factors. HPB 2013;15:170–174. 23374356 10.1111/j.1477-2574.2012.00520.x PMC3572276

[51] Burkhart RA Gerber SM Tholey RM . Incidence and severity of pancreatogenic diabetes after pancreatic resection. J Gastrointest Surg 2015;19:217–225. 25316483 10.1007/s11605-014-2669-z

[52] Lv A Qian HG Qiu H . Is central pancreatectomy truly recommendable? a 9-year single-center experience. Dig Surg 2018;35:532–538. 29275422 10.1159/000485806

[53] Song KB Kim SC Park KM . Laparoscopic central pancreatectomy for benign or low-grade malignant lesions in the pancreatic neck and proximal body. Surg Endosc 2015;29:937–946. 25149632 10.1007/s00464-014-3756-7

[54] Chen XM Zhang Y Sun DL . Laparoscopic central pancreatectomy for solid pseudopapillary tumors of the pancreas: our experience with ten cases. World J Surg Oncol 2014;12:312. 25307540 10.1186/1477-7819-12-312 PMC4210476

[55] Zhang R Xu X Yan J . Laparoscopic central pancreatectomy with pancreaticojejunostomy: preliminary experience with 8 cases. J Laparoendosc Adv Surg Tech A 2013;23:912–918. 24093934 10.1089/lap.2013.0269

[56] Senthilnathan P Gul SI Gurumurthy SS . Laparoscopic central pancreatectomy: our technique and long-term results in 14 patients. J Minim Access Surg 2015;11:167–171. 26195873 10.4103/0972-9941.158967 PMC4499920

[57] Sa Cunha A Rault A Beau C . Laparoscopic central pancreatectomy: single institution experience of 6 patients. Surgery 2007;142:405–409. 17723894 10.1016/j.surg.2007.01.035

[58] Lebedyev A Zmora O Kuriansky J . Laparoscopic distal pancreatectomy. Surg Endosc 2004;18:1427–1430. 15791363 10.1007/s00464-003-8221-y

[59] Nau P Melvin WS Narula VK . Laparoscopic distal pancreatectomy with splenic conservation: an operation without increased morbidity. Gastroenterol Res Pract 2009;2009:846340. 20049337 10.1155/2009/846340 PMC2798083

[60] Malleo G Damoli I Marchegiani G . Laparoscopic distal pancreatectomy: analysis of trends in surgical techniques, patient selection, and outcomes. Surg Endosc 2015;29:1952–1962. 25303912 10.1007/s00464-014-3890-2

[61] Bock EA Hurtuk MG Shoup M . Late complications after pancreaticoduodenectomy with pancreaticogastrostomy. J Gastrointest Surg 2012;16:914–919. 22374385 10.1007/s11605-011-1805-2

[62] Chiarelli M Gerosa M Tagliabue F . Left-sided pancreatic incidentalomas treated with laparoscopic approach: a report of 20 cases. World J Surg Oncol 2016;14:204. 27487847 10.1186/s12957-016-0949-7 PMC4973032

[63] You DD Choi SH Choi DW . Long-term effects of pancreaticoduodenectomy on glucose metabolism. ANZ J Surg 2012;82:447–451. 22571457 10.1111/j.1445-2197.2012.06080.x

[64] Müller MW Friess H Martin DJ . Long-term follow-up of a randomized clinical trial comparing Beger with pylorus-preserving Whipple procedure for chronic pancreatitis. Br J Surg 2008;95:350–356. 17933005 10.1002/bjs.5960

[65] Keck T Wellner UF Riediger H . Long-term outcome after 92 duodenum-preserving pancreatic head resections for chronic pancreatitis: comparison of Beger and Frey procedures. J Gastrointest Surg 2010;14:549–556. 20033344 10.1007/s11605-009-1119-9

[66] Riediger H Adam U Fischer E . Long-term outcome after resection for chronic pancreatitis in 224 patients. J Gastrointest Surg 2007;11:949–959. 17534689 10.1007/s11605-007-0155-6

[67] Hutchins RR Hart RS Pacifico M . Long-term results of distal pancreatectomy for chronic pancreatitis in 90 patients. Ann Surg 2002;236:612–618. 12409667 10.1097/00000658-200211000-00011 PMC1422619

[68] Falconi M Bassi C Casetti L . Long-term results of Frey’s procedure for chronic pancreatitis: a longitudinal prospective study on 40 patients. J Gastrointest Surg 2006;10:504–510. 16627215 10.1016/j.gassur.2005.09.011

[69] Sauvanet A Partensky C Sastre B . Medial pancreatectomy: a multi-institutional retrospective study of 53 patients by the French Pancreas Club. Surgery 2002;132:836–843. 12464868 10.1067/msy.2002.127552

[70] Sperti C Pasquali C Ferronato A . Median pancreatectomy for tumors of the neck and body of the pancreas. J Am Coll Surg 2000;190:711–716. 10873007 10.1016/s1072-7515(00)00286-6

[71] Sudo T Murakami Y Uemura K . Middle pancreatectomy with pancreaticogastrostomy: a technique, operative outcomes, and long-term pancreatic function. J Surg Oncol 2010;101:61–65. 19894223 10.1002/jso.21430

[72] Crippa S Bassi C Warshaw AL . Middle pancreatectomy: indications, short- and long-term operative outcomes. Ann Surg 2007;246:69–76. 17592293 10.1097/01.sla.0000262790.51512.57 PMC1899210

[73] Shikano T Nakao A Kodera Y . Middle pancreatectomy: safety and long-term results. Surgery 2010;147:21–29. 19682717 10.1016/j.surg.2009.04.036

[74] Müller MW Friess H Kleeff J . Middle segmental pancreatic resection: An option to treat benign pancreatic body lesions. Ann Surg 2006;244:909–918; discussion 18-20. 17122616 10.1097/01.sla.0000247970.43080.23 PMC1856616

[75] Machado MA Ardengh JC Makdissi FF . Minimally invasive resection of the uncinate process of the pancreas: anatomical considerations and surgical technique. Surg Innov 2022;29:600–607. 35332821 10.1177/15533506211045317

[76] Shibata S Sato T Andoh H . Outcomes and indications of segmental pancreatectomy. Comparison with distal pancreatectomy. Dig Surg 2004;21:48–53. 14707393 10.1159/000075826

[77] Yoo D Hwang S Kim KH . Pancreatic atrophy relative to external versus internal drainage of the pancreatic duct after pylorus-preserving pancreaticoduodenectomy. J Gastrointest Surg 2014;18:1604–1609. 25002021 10.1007/s11605-014-2583-4

[78] Jalleh RP Williamson RC . Pancreatic exocrine and endocrine function after operations for chronic pancreatitis. Ann Surg 1992;216:656–662. 1466619 10.1097/00000658-199212000-00007 PMC1242712

[79] Shirakawa S Matsumoto I Toyama H . Pancreatic volumetric assessment as a predictor of new-onset diabetes following distal pancreatectomy. J Gastrointest Surg 2012;16:2212–2219. 23054900 10.1007/s11605-012-2039-7 PMC3508270

[80] Goldstein MJ Toman J Chabot JA . Pancreaticogastrostomy: a novel application after central pancreatectomy. J Am Coll Surg 2004;198:871–876. 15194067 10.1016/j.jamcollsurg.2004.02.026

[81] Diener MK Huttner FJ Kieser M . Partial pancreatoduodenectomy versus duodenum-preserving pancreatic head resection in chronic pancreatitis: the multicentre, randomised, controlled, double-blind ChroPac trial. Lancet (London, England) 2017;390:1027–1037. 28901935 10.1016/S0140-6736(17)31960-8

[82] Jilesen AP van Eijck CH Busch OR . Postoperative outcomes of enucleation and standard resections in patients with a pancreatic neuroendocrine tumor. World J Surg 2016;40:715–728. 26608956 10.1007/s00268-015-3341-9 PMC4746212

[83] Hwang HK Park J Choi SH . Predicting new-onset diabetes after minimally invasive subtotal distal pancreatectomy in benign and borderline malignant lesions of the pancreas. Medicine 2017;96:e9404. 29390555 10.1097/MD.0000000000009404 PMC5758257

[84] Yoo DG Jung BH Hwang S . Prevalence analysis of de novo hepatic steatosis following pylorus-preserving pancreaticoduodenectomy. Dig Surg 2014;31:359–365. 25503526 10.1159/000368381

[85] Epelboym I Winner M DiNorcia J . Quality of life in patients after total pancreatectomy is comparable with quality of life in patients who undergo a partial pancreatic resection. J Surg Res 2014;187:189–196. 24411300 10.1016/j.jss.2013.10.004

[86] Strate T Bachmann K Busch P . Resection vs drainage in treatment of chronic pancreatitis: long-term results of a randomized trial. Gastroenterology 2008;134:1406–1411. 18471517 10.1053/j.gastro.2008.02.056

[87] Wu JM Kuo TC Yang CY . Resolution of diabetes after pancreaticoduodenectomy in patients with and without pancreatic ductal cell adenocarcinoma. Ann Surg Oncol 2013;20:242–249. 22864799 10.1245/s10434-012-2577-y

[88] Oh HM Yoon YS Han HS . Risk factors for pancreatogenic diabetes after pancreaticoduodenectomy. Korean J Hepatobiliary Pancreat Surg 2012;16:167–171. 26388929 10.14701/kjhbps.2012.16.4.167 PMC4575001

[89] Maignan A Ouaissi M Turrini O . Risk factors of exocrine and endocrine pancreatic insufficiency after pancreatic resection: a multi-center prospective study. J Visc Surg 2018;155:173–181. 29396112 10.1016/j.jviscsurg.2017.10.007

[90] Jiang Y Jin JB Zhan Q . Robot-assisted duodenum-preserving pancreatic head resection with pancreaticogastrostomy for benign or premalignant pancreatic head lesions: a single-centre experience. Int J Med Rob + Computer Assist Surg 2018;14:e1903. 10.1002/rcs.1903 29498195

[91] Abood GJ Can MF Daouadi M . Robotic-assisted minimally invasive central pancreatectomy: technique and outcomes. J Gastrointest Surg 2013;17:1002–1008. 23325340 10.1007/s11605-012-2137-6

[92] Shimada K Sakamoto Y Esaki M . Role of medial pancreatectomy in the management of intraductal papillary mucinous neoplasms and islet cell tumors of the pancreatic neck and body. Dig Surg 2008;25:46–51. 18292661 10.1159/000117823

[93] Keck T Adam U Makowiec F . Short- and long-term results of duodenum preservation versus resection for the management of chronic pancreatitis: a prospective, randomized study. Surgery 2012;152(3 Suppl 1):S95–S102. 22906892 10.1016/j.surg.2012.05.016

[94] Tang CW Feng WM Bao Y . Spleen-preserving distal pancreatectomy or distal pancreatectomy with splenectomy?: perioperative and patient-reported outcome analysis. J Clin Gastroenterol 2014;48:e62–e66. 24231937 10.1097/MCG.0000000000000021 PMC4222703

[95] Balzano G Zerbi A Veronesi P . Surgical treatment of benign and borderline neoplasms of the pancreatic body. Dig Surg 2003;20:506–510. 14506331 10.1159/000073646

[96] Lim PW Dinh KH Sullivan M . Thirty-day outcomes underestimate endocrine and exocrine insufficiency after pancreatic resection. HPB 2016;18:360–366. 27037206 10.1016/j.hpb.2015.11.003 PMC4814621

[97] Govil S Imrie CW . Value of splenic preservation during distal pancreatectomy for chronic pancreatitis. Br J Surg 1999;86:895–898. 10417561 10.1046/j.1365-2168.1999.01179.x

[98] Mijatovic-Vukas J Capling L Cheng S . Associations of diet and physical activity with risk for gestational diabetes mellitus: a systematic review and meta-analysis. Nutrients 2018;10:698. 29849003 10.3390/nu10060698 PMC6024719

[99] Lee Y Park K . Adherence to a vegetarian diet and diabetes risk: a systematic review and meta-analysis of observational studies. Nutrients 2017;9:603. 28613258 10.3390/nu9060603 PMC5490582

[100] Takeuchi M Okamoto K Takagi T . Ethnic difference in patients with type 2 diabetes mellitus in inter-East Asian populations: a systematic review and meta-analysis focusing on fasting serum insulin. Diabetes Res Clin Pract 2008;81:370–376. 18649967 10.1016/j.diabres.2008.05.013

[101] Jenum AK Brekke I Mdala I . Effects of dietary and physical activity interventions on the risk of type 2 diabetes in South Asians: meta-analysis of individual participant data from randomised controlled trials. Diabetologia 2019;62:1337–1348. 31201437 10.1007/s00125-019-4905-2

[102] Martín-Peláez S Fito M Castaner O . Mediterranean diet effects on type 2 diabetes prevention, disease progression, and related mechanisms. a review. Nutrients 2020;12:2236. 32726990 10.3390/nu12082236 PMC7468821

[103] Atun R de Andrade LO Almeida G . Health-system reform and universal health coverage in Latin America. Lancet (London, England) 2015;385:1230–1247. 25458725 10.1016/S0140-6736(14)61646-9

[104] Jiang L Ning D Chen XP . Improvement in distal pancreatectomy for tumors in the body and tail of the pancreas. World J Surg Oncol 2021;19:49. 33588845 10.1186/s12957-021-02159-9 PMC7885351

[105] Zhang T Du X Zhao Y . Laparoscopic surgery for pancreatic lesions: current status and future. Front Med 2011;5:277–282. 21964709 10.1007/s11684-011-0147-5

[106] Teich T Zaharieva DP Riddell MC . Advances in exercise, physical activity, and diabetes mellitus. Diabetes Technol Ther 2019;21(S1):S112–s22. 30785316 10.1089/dia.2019.2509

[107] Meara JG Leather AJ Hagander L . Global Surgery 2030: evidence and solutions for achieving health, welfare, and economic development. Lancet (London, England) 2015;386:569–624. 25924834 10.1016/S0140-6736(15)60160-X

[108] Vollmer CM Jr Sanchez N Gondek S . A root-cause analysis of mortality following major pancreatectomy. J Gastrointest Surg 2012;16:89–102. 22065319 10.1007/s11605-011-1753-x

[109] Jamal A Shakeel O Mohsin J . Pancreaticoduodenectomy: outcomes of a complex surgical procedure from a developing country. Pancreatology 2020;20:1534–1539. 32928685 10.1016/j.pan.2020.08.013

[110] Malka D Hammel P Sauvanet A . Risk factors for diabetes mellitus in chronic pancreatitis. Gastroenterology 2000;119:1324–1332. 11054391 10.1053/gast.2000.19286

[111] Liu J Knezetic JA Strömmer L . The intracellular mechanism of insulin resistance in pancreatic cancer patients. J Clin Endocrinol Metab 2000;85:1232–1238. 10720068 10.1210/jcem.85.3.6400

[112] Gomez-Chou SB Swidnicka-Siergiejko AK Badi N . Lipocalin-2 promotes pancreatic ductal adenocarcinoma by regulating inflammation in the tumor microenvironment. Cancer Res 2017;77:2647–2660. 28249896 10.1158/0008-5472.CAN-16-1986 PMC5441230

[113] Mezza T Moffa S Ferraro PM . Bile modulates secretion of incretins and insulin: a study of human extrahepatic cholestasis. J Clin Endocrinol Metab 2019;104:2685–2694. 30874733 10.1210/jc.2018-02804

[114] Pour PM Permert J Mogaki M . Endocrine aspects of exocrine cancer of the pancreas. Their patterns and suggested biologic significance. Am J Clin Pathol 1993;100:223–230. 8379530 10.1093/ajcp/100.3.223

[115] Wang X Misawa R Zielinski MC . Regional differences in islet distribution in the human pancreas--preferential beta-cell loss in the head region in patients with type 2 diabetes. PLoS ONE 2013;8:e67454. 23826303 10.1371/journal.pone.0067454 PMC3691162

[116] Muscogiuri G Mezza T Prioletta A . Removal of duodenum elicits GLP-1 secretion. Diabetes Care 2013;36:1641–1646. 23393218 10.2337/dc12-0811 PMC3661831

[117] Korner J Bessler M Inabnet W . Exaggerated glucagon-like peptide-1 and blunted glucose-dependent insulinotropic peptide secretion are associated with Roux-en-Y gastric bypass but not adjustable gastric banding. Surg Obes Relat Dis 2007;3:597–601. 17936091 10.1016/j.soard.2007.08.004 PMC2134840

[118] Mathur A Pitt HA Marine M . Fatty pancreas: a factor in postoperative pancreatic fistula. Ann Surg 2007;246:1058–1064. 18043111 10.1097/SLA.0b013e31814a6906

[119] Seymour NE Brunicardi FC Chaiken RL . Reversal of abnormal glucose production after pancreatic resection by pancreatic polypeptide administration in man. Surgery 1988;104:119–129. 3041640

[120] Hart PA Bellin MD Andersen DK . Type 3c (pancreatogenic) diabetes mellitus secondary to chronic pancreatitis and pancreatic cancer. Lancet Gastroenterol Hepatol 2016;1:226–237. 28404095 10.1016/S2468-1253(16)30106-6 PMC5495015

