# Supplementary material for: Mapping global new-onset, worsening, and resolution of diabetes following partial pancreatectomy: a systematic review and meta-analysis
Source: Int J Surg. 2023 Dec 21;110(3):1770–80. doi: 10.1097/JS9.0000000000000998 (PMC10942179; doi:10.1097/JS9.0000000000000998)
Supplement: SUPPLEMENTARY MATERIAL [file js9-110-1770-s003.doc]

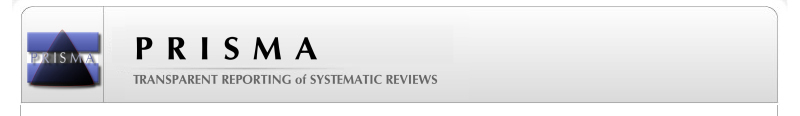
**PRISMA 2009 Flow Diagram**

**Screening**

**Included**

**Eligibility**

**Identification**

Records identified through database searching
(n = 8736)

Additional records identified through other sources
(n = 0)

Records after duplicates removed
(n = 5259)

Records screened
(n = 5259)

Records excluded
(n = 5116)

Full-text articles assessed for eligibility
(n = 143)

Full-text articles excluded, with reasons
(n = 61)

38 non-relevant or duplicate data

7 total pancreatectomy

1 individuals < 18 years

15 without the exact number of pre- and postoperative diabetes

Studies included in qualitative synthesis
(n = 82)

Studies included in quantitative synthesis (meta-analysis)
(n = 82)
