# Supplementary material for: Mapping global new-onset, worsening, and resolution of diabetes following partial pancreatectomy: a systematic review and meta-analysis
Source: Int J Surg. 2023 Dec 21;110(3):1770–80. doi: 10.1097/JS9.0000000000000998 (PMC10942179; doi:10.1097/JS9.0000000000000998)

**Supplementary materials to**

**Mapping** **global new-onset, worsening, and resolution of diabetes following partial pancreatectomy: A systematic review and meta-analysis**

**Supplementary methods. Searching strategy for global new-onset, worsening, and resolution of diabetes following partial pancreatectomy.**

| **Database searched** | **via** | **Records** |
| --- | --- | --- |
| PubMed | pubmed.ncbi.nlm.nih.gov | 2746 |
| Embase | Ovid | 4093 |
| Cochrane Library | Wiley | 107 |
| Web of Science Core Collection | Web of Knowledge | 1790 |
| **Total** |  | **8736** |

| **Database** | **Searching strategy** |
| --- | --- |
| **PubMed** |  |
| **#1** | "Pancreatectomy"[Mesh] OR ("pancreatectomy" OR "pancreatoduodenectomy" OR "duodenum-preserving pancreatic head resection" OR "pancreatic head resection with segmental duodenectomy" OR "distal pancreatectomy" OR "pancreatic left resection" OR "central pancreatectomy" OR “spleno-pancreatectomy” OR "pancreatic middle segment resection" OR "tumor enucleation") |
| **#2** | "Diabetes Mellitus"[Mesh] OR ("diabetes" OR "endocrine insufficiency") |
| **#3** | #1 AND #2 (2746) |
| **Embase** |  |
| **#1** | exp pancreatectomy/ or ("pancreatectomy" or "pancreatoduodenectomy" or "duodenum-preserving pancreatic head resection" or "pancreatic head resection with segmental duodenectomy" or "distal pancreatectomy" or "pancreatic left resection" or “spleno-pancreatectomy” or "central pancreatectomy" or "pancreatic middle segment resection" or "tumor enucleation").af. |
| **#2** | exp diabetes mellitus/ or ("diabetes" or "endocrine insufficiency").af. |
| **#3** | #1 AND #2 (4093) |
| **Cochrane Library** |  |
| **#1** | "pancreatectomy" OR "pancreatoduodenectomy" OR "duodenum-preserving pancreatic head resection" OR "pancreatic head resection with segmental duodenectomy" OR "distal pancreatectomy" OR "pancreatic left resection" OR “spleno-pancreatectomy” OR "central pancreatectomy" OR "pancreatic middle segment resection" OR "tumor enucleation" |
| **#2** | "diabetes" OR "endocrine insufficiency" |
| **#3** | #1 AND #2 (107) |
| **Web of Science** |  |
| **#1** | "pancreatectomy" OR "pancreatoduodenectomy" OR "duodenum-preserving pancreatic head resection" OR "pancreatic head resection with segmental duodenectomy" OR "distal pancreatectomy" OR "pancreatic left resection" OR “spleno-pancreatectomy” OR "central pancreatectomy" OR "pancreatic middle segment resection" OR "tumor enucleation" |
| **#2** | "diabetes" OR "endocrine insufficiency" |
| **#3** | #1 AND #2 (1790) |

**Supplementary Table 1. Characteristics of included studies for meta-analysis.**

**(A) Characteristics of included studies for new-onset diabetes.**

| Study | Country/Area | Publication year | Study period | New-onset DM | Patients | DM diagnosis | Study Quality |
| --- | --- | --- | --- | --- | --- | --- | --- |
| DiNorcia, J. ^[16]^ | USA | 2010 | 1997-2009 | 21 | 84 | Need for blood glucose control measures | 9 |
| Hirono, S. ^[17]^ | Japan | 2009 | 1999-2008 | 7 | 44 | Need for blood glucose control measures | 7 |
| Paiella, S. ^[18]^ | Italy | 2019 | 1990-2017 | 6 | 75 | Telephone interview/Out-patient consultation | 8 |
| Brown, K. M. ^[19]^ | USA | 2006 | 1999-2004 | 0 | 9 | NR | 6 |
| Lee, D. H. ^[20]^ | Korea | 2020 | 2000-2015 | 20 | 124 | FBG/OGTT | 8 |
| Dumitrascu, T. ^[21]^ | Romania | 2012 | 2002-2012 | 3 | 42 | FBG | 7 |
| Adham, M. ^[22]^ | France | 2008 | 1987-2005 | 0 | 49 | NR | 7 |
| Wu, J. M. ^[23]^ | China | 2018 | 2001-2010 | 312 | 1410 | NR | 7 |
| Litwin, J. ^[24]^ | Poland | 2008 | 2005-2006 | 3 | 14 | FBG/OGTT | 7 |
| Sato, N. ^[25]^ | Japan | 1998 | 1992-1997 | 3 | 17 | WHO-criteria | 7 |
| Lee, S. E. ^[26]^ | Korea | 2010 | 1995-2007 | 28 | 256 | NR | 7 |
| Dumitrascu, T. ^[27]^ | Romania | 2014 | 2000-2012 | 11 | 62 | FBG | 7 |
| Ocuin, L. M. ^[28]^ | USA | 2008 | 2000-2006 | 9 | 23 | FBG/OGTT | 7 |
| Zhang, R. C. ^[29]^ | China | 2017 | 1997-2015 | 3 | 33 | Telephone interview/Out-patient consultation | 7 |
| Li, Y. ^[30]^ | China | 2017 | 2008-2014 | 16 | 132 | FBG/OGTT | 6 |
| Fujii, T. ^[31]^ | Japan | 2011 | 1991-2009 | 9 | 109 | NR | 7 |
| Jang, J. Y. ^[32]^ | Korea | 2002 | 1998-1999 | 10 | 28 | FBG/OGTT | 7 |
| Sakata, N. ^[33]^ | Japan | 2011 | 2008 | 2 | 25 | FBG/OGTT | 7 |
| Tariq, M. ^[34]^ | USA | 2020 | 2007-2017 | 37 | 131 | WHO-criteria | 8 |
| Lee, C. Y. C. ^[35]^ | Australia | 2020 | 2011-2017 | 11 | 95 | WHO-criteria | 7 |
| Mori, Y. ^[36]^ | Japan | 2012 | 2009-2010 | 1 | 14 | OGTT | 6 |
| Siegel, J. B. ^[37]^ | USA | 2022 | 2012-2020 | 26 | 76 | Need for blood glucose control measures | 7 |
| King, J. ^[38]^ | USA | 2008 | 1992-2006 | 10 | 111 | Need for blood glucose control measures | 7 |
| Yun, S. P. ^[39]^ | Korea | 2017 | 2007-2012 | 16 | 66 | FBG/OGTT/Need for blood glucose control measures | 6 |
| Izbicki, J. R. ^[40]^ | Germany | 1995 | 1992 | 0 | 25 | OGTT | 6 |
| Takada, T. ^[41]^ | Russia | 2003 | <2003 | 2 | 49 | NR | 6 |
| Belyaev, O. ^[42]^ | Germany | 2013 | 2009 | 6 | 58 | Need for blood glucose control measures | 7 |
| Wang, Z. Z. ^[43]^ | China | 2019 | 2017 | 0 | 11 | NR | 7 |
| Cataldegirmen, G. ^[44]^ | Germany | 2010 | 1992-2007 | 15 | 98 | OGTT | 8 |
| Orfanidis, N. T. ^[45]^ | USA | 2012 | 2006-2010 | 4 | 32 | Telephone interview | 7 |
| van der Gaag, N. A. ^[46]^ | Netherlands | 2012 | 1992-2006 | 23 | 78 | FBG/Need for blood glucose control measures | 8 |
| Lemaire, E. ^[47]^ | France | 2000 | 1987-1996 | 0 | 17 | WHO-criteria | 8 |
| Wu, J. M. ^[48]^ | China | 2015 | 2000-2011 | 632 | 3914 | WHO-criteria | 9 |
| Hamilton, L. ^[49]^ | USA | 2007 | 1998-2004 | 10 | 27 | Need for blood glucose control measures | 7 |
| Ferrara, M. J. ^[50]^ | USA | 2013 | 2004-2010 | 22 | 564 | Need for blood glucose control measures | 8 |
| Burkhart, R. A. ^[51]^ | USA | 2015 | 2006-2013 | 41 | 190 | Need for blood glucose control measures | 9 |
| Lv, A. ^[52]^ | China | 2018 | 2007-2014 | 2 | 38 | WHO-criteria | 8 |
| Song, K. B. ^[53]^ | Korea | 2015 | 2007-2010 | 26 | 118 | Need for blood glucose control measures | 9 |
| Chen, X. M. ^[54]^ | China | 2014 | 2009-2013 | 0 | 10 | FBG | 7 |
| Zhang, R. ^[55]^ | China | 2013 | 2011-2013 | 0 | 8 | FBG | 7 |
| Senthilnathan, P. ^[56]^ | India | 2015 | 2004-2013 | 2 | 14 | FBG/HbA1c | 7 |
| Sa Cunha, A. ^[57]^ | France | 2007 | 1999-2006 | 0 | 6 | WHO-criteria | 8 |
| Lebedyev, A. ^[58]^ | Israel | 2004 | 1997-2003 | 2 | 12 | NR | 7 |
| Nau, P. ^[59]^ | USA | 2009 | 1999-2009 | 5 | 24 | Need for blood glucose control measures | 8 |
| Malleo, G. ^[60]^ | USA | 2015 | 1999-2012 | 12 | 90 | FBG/OGTT | 8 |
| Bock, E. A. ^[61]^ | USA | 2012 | 1995-2010 | 18 | 77 | HbA1c | 9 |
| Chiarelli, M. ^[62]^ | Italy | 2016 | 2010-2015 | 5 | 20 | NR | 9 |
| You, D. D. ^[63]^ | Korea | 2012 | 2003-2004 | 9 | 43 | FBG/OGTT/Need for blood glucose control measures | 8 |
| Müller, M. W. ^[64]^ | Germany | 2008 | 1991-1993 | 10 | 20 | FBG/HbA1c/Need for blood glucose control measures | 6 |
| Keck, T. ^[65]^ | Germany | 2010 | 1996-2007 | 24 | 62 | WHO-criteria | 7 |
| Riediger, H. ^[66]^ | Germany | 2007 | 1994-2005 | 56 | 160 | WHO-criteria | 8 |
| Hutchins, R. R. ^[67]^ | UK | 2002 | 1980-2000 | 32 | 69 | OGTT | 8 |
| Falconi, M. ^[68]^ | Italy | 2006 | 1997-2001 | 1 | 30 | OGTT | 8 |
| Sauvanet, A. ^[69]^ | France | 2002 | 1990-1998 | 2 | 46 | NR | 8 |
| Sperti, C. ^[70]^ | USA | 2000 | 1985-1998 | 0 | 10 | OGTT | 8 |
| Sudo, T. ^[71]^ | Japan | 2010 | 1996-2008 | 5 | 17 | Need for blood glucose control measures | 9 |
| Crippa, S. ^[72]^ | USA | 2007 | 1990-2005 | 16 | 131 | WHO-criteria | 8 |
| Shikano, T. ^[73]^ | Japan | 2010 | 1991-2006 | 13 | 107 | FBG/HbA2c | 7 |
| Müller, M. W. ^[74]^ | Germany | 2006 | 2001-2005 | 16 | 97 | FBG/HbA2c | 8 |
| Machado, M. A. ^[75]^ | Brazil | 2022 | 2008-2019 | 0 | 29 | NR | 7 |
| Shibata, S. ^[76]^ | Japan | 2004 | 1989-2002 | 3 | 17 | OGTT | 8 |
| Yoo, D. ^[77]^ | Korea | 2014 | 2009-2011 | 1 | 46 | NR | 7 |
| Jalleh, R. P. ^[78]^ | UK | 1992 | 1978-1991 | 23 | 76 | FBG/OGTT | 7 |
| Shirakawa, S. ^[79]^ | Japan | 2012 | 2005-2011 | 22 | 61 | WHO-criteria | 8 |
| Goldstein, M. J. ^[80]^ | USA | 2004 | 1999-2002 | 0 | 12 | HbA1c | 8 |
| Diener, M. K. ^[81]^ | Germany | 2017 | 2009-2013 | 7 | 162 | WHO-criteria | 6 |
| Jilesen, A. P. ^[82]^ | Netherlands | 2016 | 1992-2013 | 25 | 189 | WHO-criteria | 8 |
| Hwang, H. K. ^[83]^ | Korea | 2017 | 2005-2014 | 9 | 34 | WHO-criteria | 6 |
| Yoo, D. G. ^[84]^ | Korea | 2014 | 2008-2009 | 3 | 81 | NR | 6 |
| Epelboym, I. ^[85]^ | USA | 2014 | 1994-2011 | 1 | 7 | NR | 6 |
| Strate, T. ^[86]^ | Germany | 2008 | 1995-1997 | 28 | 46 | OGTT | 6 |
| Wu, J. M. ^[87]^ | China | 2013 | 2005-2011 | 26 | 323 | FBG | 8 |
| Oh, H. M. ^[88]^ | Korea | 2012 | 2003-2009 | 17 | 98 | FBG/OGTT/RBG/Presence of diabetes symptoms | 8 |
| Maignan, A. ^[89]^ | France | 2018 | 2014-2015 | 7 | 70 | FBG | 7 |
| Jiang, Y. ^[90]^ | China | 2018 | 2010-2016 | 4 | 59 | Need for blood glucose control measures | 7 |
| Abood, G. J. ^[91]^ | USA | 2013 | 2009-2010 | 0 | 6 | Need for blood glucose control measures | 7 |
| Shimada, K. ^[92]^ | Japan | 2008 | 2000-2007 | 0 | 12 | Need for blood glucose control measures | 6 |
| Keck, T. ^[93]^ | Germany | 2012 | 1997-2001 | 18 | 65 | OGTT | 6 |
| Tang, C. W. ^[94]^ | China | 2014 | 2005-2009 | 12 | 137 | Telephone interview | 6 |
| Balzano, G. ^[95]^ | Italy | 2003 | 1999-2000 | 6 | 63 | Telephone interview | 6 |
| Lim, P. W. ^[96]^ | USA | 2016 | 2002-2012 | 28 | 178 | Need for blood glucose control measures | 8 |
| Govil, S. ^[97]^ | UK | 1999 | 1982-1998 | 12 | 32 | NR | 7 |

**(B) Characteristics of included studies for worsening diabetes.**

| Study | Country/Area | Publication year | Study period | Worsening DM | Patients | Definition of worsening DM | Study Quality |
| --- | --- | --- | --- | --- | --- | --- | --- |
| DiNorcia, J. ^[16]^ | USA | 2010 | 1997-2009 | 9 | 16 | Deterioration in the metabolic control | 9 |
| Hirono, S. ^[17]^ | Japan | 2009 | 1999-2008 | 3 | 3 | Deterioration in the metabolic control | 7 |
| Paiella, S. ^[18]^ | Italy | 2019 | 1990-2017 | 0 | 44 | Telephone interview/Out-patient consultation | 8 |
| Brown, K. M. ^[19]^ | USA | 2006 | 1999-2004 | 0 | 1 | NR | 6 |
| Lee, D. H. ^[20]^ | Korea | 2020 | 2000-2015 | 6 | 41 | Deterioration in the metabolic control | 8 |
| Dumitrascu, T. ^[21]^ | Romania | 2012 | 2002-2012 | 1 | 1 | NR | 7 |
| Wu, J. M. ^[23]^ | China | 2018 | 2001-2010 | 173 | 289 | Deterioration in the metabolic control | 7 |
| Lee, S. E. ^[26]^ | Korea | 2010 | 1995-2007 | 0 | 46 | NR | 7 |
| Dumitrascu, T. ^[27]^ | Romania | 2014 | 2000-2012 | 2 | 2 | NR | 7 |
| Zhang, R. C. ^[29]^ | China | 2017 | 1997-2015 | 0 | 2 | Telephone interview/Out-patient consultation | 7 |
| Izbicki, J. R. ^[40]^ | Germany | 1995 | 1992 | 2 | 17 | Deterioration in the metabolic control | 6 |
| Orfanidis, N. T. ^[45]^ | USA | 2012 | 2006-2010 | 4 | 9 | Deterioration in the metabolic control | 7 |
| Lemaire, E. ^[47]^ | France | 2000 | 1987-1996 | 2 | 2 | Deterioration in the metabolic control | 8 |
| Burkhart, R. A. ^[51]^ | USA | 2015 | 2006-2013 | 27 | 67 | Deterioration in the metabolic control | 9 |
| Lv, A. ^[52]^ | China | 2018 | 2007-2014 | 0 | 2 | Deterioration in the metabolic control | 8 |
| You, D. D. ^[63]^ | Korea | 2012 | 2003-2004 | 10 | 12 | Deterioration in the metabolic control | 8 |
| Hutchins, R. R. ^[67]^ | UK | 2002 | 1980-2000 | 4 | 8 | Deterioration in the metabolic control | 8 |
| Falconi, M. ^[68]^ | Italy | 2006 | 1997-2001 | 2 | 10 | Deterioration in the metabolic control | 8 |
| Sauvanet, A. ^[69]^ | France | 2002 | 1990-1998 | 1 | 2 | NR | 8 |
| Crippa, S. ^[72]^ | USA | 2007 | 1990-2005 | 5 | 14 | Deterioration in the metabolic control | 8 |
| Jalleh, R. P. ^[78]^ | UK | 1992 | 1978-1991 | 3 | 4 | NR | 7 |
| Maignan, A. ^[89]^ | France | 2018 | 2014-2015 | 7 | 21 | Deterioration in the metabolic control | 7 |
| Jiang, Y. ^[90]^ | China | 2018 | 2010-2016 | 2 | 7 | Deterioration in the metabolic control | 7 |
| Tang, C. W. ^[94]^ | China | 2014 | 2005-2009 | 13 | 23 | Telephone interview | 6 |
| Govil, S. ^[97]^ | UK | 1999 | 1982-1998 | 1 | 4 | NR | 7 |

**(C) Characteristics of included studies for resolution of diabetes.**

| Study | Country/Area | Publication year | Study period | Resolution of DM | Patients | Definition of resolution of DM | Study Quality |
| --- | --- | --- | --- | --- | --- | --- | --- |
| Litwin, J. ^[24]^ | Poland | 2008 | 2005-2006 | 2 | 4 | Recovery from previously diagnosed DM | 7 |
| Sato, N. ^[25]^ | Japan | 1998 | 1992-1997 | 7 | 17 | Glucose tolerance improvement | 7 |
| Sakata, N. ^[33]^ | Japan | 2011 | 2008 | 2 | 7 | Recovery from previously diagnosed DM | 7 |
| Tariq, M. ^[34]^ | USA | 2020 | 2007-2017 | 14 | 85 | Recovery from previously diagnosed DM | 8 |
| Mori, Y. ^[36]^ | Japan | 2012 | 2009-2010 | 11 | 20 | Recovery from previously diagnosed DM | 6 |
| Izbicki, J. R. ^[40]^ | Germany | 1995 | 1992 | 3 | 17 | Remarkable improvement of previously diabetic status (saving 16, 20, and 24 international units of insulin per day) | 6 |
| Wu, J. M. ^[48]^ | China | 2015 | 2000-2011 | 174 | 861 | Recovery from previously diagnosed DM | 9 |
| Burkhart, R. A. ^[51]^ | USA | 2015 | 2006-2013 | 5 | 67 | A reduction in the number of oral medications taken or a change from insulin dependence to oral medications alone | 9 |
| Müller, M. W. ^[64]^ | Germany | 2008 | 1991-1993 | 1 | 9 | Recovery from previously diagnosed DM | 6 |
| Falconi, M. ^[68]^ | Italy | 2006 | 1997-2001 | 4 | 10 | A change from insulin dependence to oral medications alone | 8 |
| Sauvanet, A. ^[69]^ | France | 2002 | 1990-1998 | 1 | 2 | Recovery from previously diagnosed DM | 8 |
| Wu, J. M. ^[87]^ | China | 2013 | 2005-2011 | 30 | 133 | Recovery from previously diagnosed DM | 8 |
| Shimada, K. ^[92]^ | Japan | 2008 | 2000-2007 | 1 | 1 | Antidiabetic drugs or insulin therapy no longer obligatory | 6 |

**Supplementary Table 2. Sensitivity analysis for detecting outliers by using leave-one-out function.**

|  | resid | se | z |
| --- | --- | --- | --- |
| 71 | 2.1290 | 0.8070 | 2.6380 |
| 31 | 2.1143 | 0.8219 | 2.5725 |
| 35 | -1.5734 | 0.7904 | -1.9907 |
| 52 | 1.5395 | 0.8082 | 1.9049 |
| 49 | 1.6831 | 0.8952 | 1.8801 |
| 7 | -2.9447 | 1.6225 | -1.8148 |
| 62 | -2.1588 | 1.2774 | -1.6900 |
| 66 | -1.4583 | 0.8636 | -1.6887 |
| 69 | -1.6154 | 0.9740 | -1.6585 |
| 60 | -2.4243 | 1.6280 | -1.4891 |
| 50 | 1.2223 | 0.8248 | 1.4819 |
| 26 | -1.5090 | 1.0644 | -1.4177 |
| 25 | -2.2779 | 1.6300 | -1.3975 |
| 13 | 1.2381 | 0.8943 | 1.3845 |
| 54 | -1.4422 | 1.0658 | -1.3531 |
| 82 | 1.1693 | 0.8666 | 1.3494 |
| 64 | 1.1082 | 0.8297 | 1.3356 |
| 53 | -1.7155 | 1.2848 | -1.3353 |
| 51 | 1.0621 | 0.8029 | 1.3228 |
| 34 | 1.1489 | 0.8821 | 1.3025 |
| 22 | 1.0262 | 0.8237 | 1.2458 |
| 17 | 1.0913 | 0.8812 | 1.2384 |
| 32 | -1.8994 | 1.6362 | -1.1609 |
| 37 | -1.2389 | 1.0702 | -1.1576 |
| 75 | -0.9694 | 0.9422 | -1.0289 |
| 63 | 0.8432 | 0.8298 | 1.0161 |
| 72 | -0.7849 | 0.8141 | -0.9642 |
| 77 | -1.5613 | 1.6438 | -0.9498 |
| 65 | -1.5613 | 1.6438 | -0.9498 |
| 6 | -0.9112 | 0.9905 | -0.9199 |
| 19 | 0.7449 | 0.8164 | 0.9124 |
| 28 | -1.4775 | 1.6460 | -0.8976 |
| 3 | -0.7891 | 0.8968 | -0.8799 |
| 16 | -0.7550 | 0.8628 | -0.8751 |
| 78 | 0.7165 | 0.8406 | 0.8523 |
| 55 | -1.3861 | 1.6487 | -0.8407 |
| 39 | -1.3861 | 1.6487 | -0.8407 |
| 56 | 0.7991 | 0.9551 | 0.8366 |
| 79 | -0.6897 | 0.8463 | -0.8150 |
| 4 | -1.2855 | 1.6518 | -0.7782 |
| 23 | -0.6579 | 0.8578 | -0.7670 |
| 68 | 0.6528 | 0.8845 | 0.7381 |
| 18 | -0.7854 | 1.0810 | -0.7265 |
| 40 | -1.1737 | 1.6557 | -0.7089 |
| 21 | -0.9065 | 1.3044 | -0.6949 |
| 1 | 0.5761 | 0.8345 | 0.6903 |
| 80 | -0.5947 | 0.9011 | -0.6599 |
| 14 | -0.6451 | 0.9968 | -0.6472 |
| 24 | 0.5344 | 0.8461 | 0.6316 |
| 74 | -0.5399 | 0.8875 | -0.6084 |
| 47 | 0.5738 | 0.9479 | 0.6053 |
| 46 | 0.4862 | 0.8407 | 0.5783 |
| 27 | -0.5013 | 0.9030 | -0.5551 |
| 76 | -0.9042 | 1.6669 | -0.5425 |
| 42 | -0.9042 | 1.6669 | -0.5425 |
| 11 | -0.4391 | 0.8195 | -0.5359 |
| 8 | 0.4150 | 0.8002 | 0.5186 |
| 38 | 0.4088 | 0.8276 | 0.4940 |
| 36 | 0.3820 | 0.8169 | 0.4676 |
| 20 | -0.3732 | 0.8574 | -0.4352 |
| 48 | 0.3416 | 0.8807 | 0.3878 |
| 15 | -0.3205 | 0.8395 | -0.3818 |
| 58 | -0.3177 | 0.8491 | -0.3742 |
| 57 | -0.3117 | 0.8396 | -0.3712 |
| 9 | 0.3700 | 1.0278 | 0.3600 |
| 44 | 0.3348 | 0.9415 | 0.3556 |
| 30 | -0.2840 | 0.9579 | -0.2965 |
| 67 | -0.2187 | 0.8256 | -0.2649 |
| 45 | -0.2092 | 0.8550 | -0.2447 |
| 12 | 0.1341 | 0.8642 | 0.1551 |
| 73 | 0.1065 | 0.8416 | 0.1266 |
| 61 | 0.1263 | 1.0185 | 0.1240 |
| 10 | 0.1263 | 1.0185 | 0.1240 |
| 41 | -0.1278 | 1.1018 | -0.1160 |
| 70 | -0.1277 | 1.3397 | -0.0954 |
| 29 | -0.0455 | 0.8457 | -0.0538 |
| 59 | 0.0449 | 0.8437 | 0.0532 |
| 43 | 0.0563 | 1.1095 | 0.0507 |
| 33 | 0.0193 | 0.8002 | 0.0241 |
| 5 | 0.0177 | 0.8348 | 0.0212 |
| 81 | -0.0125 | 0.8245 | -0.0152 |
| 2 | 0.0008 | 0.8973 | 0.0009 |

|  | estimate | zval | pval | ci.lb | ci.ub | Q | Qp | tau^2^ | I^2^ | H^2^ |
| --- | --- | --- | --- | --- | --- | --- | --- | --- | --- | --- |
| 1 | 0.157803 | -15.875106 | 0 | 0.132225 | 0.187260 | 493.046781 | 0 | 0.621693 | 90.205667 | 10.209986 |
| 2 | 0.158981 | -15.784978 | 0 | 0.133233 | 0.188622 | 495.449543 | 0 | 0.624064 | 90.323204 | 10.333999 |
| 3 | 0.160668 | -15.785855 | 0 | 0.134874 | 0.190310 | 490.688805 | 0 | 0.612175 | 90.156930 | 10.159432 |
| 4 | 0.159904 | -15.915139 | 0 | 0.134326 | 0.189289 | 494.619171 | 0 | 0.612396 | 90.203476 | 10.207703 |
| 5 | 0.158906 | -15.753200 | 0 | 0.133112 | 0.188610 | 495.235047 | 0 | 0.626018 | 90.258026 | 10.264860 |
| 6 | 0.160605 | -15.819873 | 0 | 0.134864 | 0.190179 | 492.444565 | 0 | 0.611230 | 90.166755 | 10.169583 |
| 7 | 0.161050 | -15.941693 | 0 | 0.135480 | 0.190383 | 490.866727 | 0 | 0.601747 | 90.047195 | 10.047419 |
| 8 | 0.157998 | -15.816747 | 0 | 0.132327 | 0.187572 | 478.559454 | 0 | 0.624948 | 88.239970 | 8.503380 |
| 9 | 0.158526 | -15.874403 | 0 | 0.132926 | 0.187987 | 495.515700 | 0 | 0.621042 | 90.310695 | 10.320658 |
| 10 | 0.158852 | -15.844188 | 0 | 0.133197 | 0.188375 | 495.606632 | 0 | 0.621579 | 90.317300 | 10.327698 |
| 11 | 0.160031 | -15.725400 | 0 | 0.134162 | 0.189794 | 486.558863 | 0 | 0.620394 | 90.108068 | 10.109249 |
| 12 | 0.158692 | -15.785143 | 0 | 0.132953 | 0.188332 | 495.600147 | 0 | 0.625215 | 90.312489 | 10.322569 |
| 13 | 0.157098 | -16.101849 | 0 | 0.131876 | 0.186110 | 489.454668 | 0 | 0.606284 | 90.071150 | 10.071660 |
| 14 | 0.160103 | -15.808508 | 0 | 0.134359 | 0.189699 | 493.850104 | 0 | 0.615950 | 90.235255 | 10.240923 |
| 15 | 0.159694 | -15.734917 | 0 | 0.133849 | 0.189437 | 492.324023 | 0 | 0.622438 | 90.230705 | 10.236153 |
| 16 | 0.160715 | -15.771631 | 0 | 0.134898 | 0.190386 | 488.763115 | 0 | 0.612369 | 90.136161 | 10.138041 |
| 17 | 0.157219 | -16.051177 | 0 | 0.131919 | 0.186329 | 490.238803 | 0 | 0.609968 | 90.116983 | 10.118367 |
| 18 | 0.160168 | -15.834831 | 0 | 0.134462 | 0.189712 | 493.974252 | 0 | 0.614221 | 90.218053 | 10.222914 |
| 19 | 0.157460 | -15.934122 | 0 | 0.131981 | 0.186800 | 486.900211 | 0 | 0.617740 | 90.055818 | 10.056131 |
| 20 | 0.159795 | -15.745220 | 0 | 0.133964 | 0.189518 | 492.828417 | 0 | 0.621230 | 90.250624 | 10.257067 |
| 21 | 0.159971 | -15.877862 | 0 | 0.134335 | 0.189429 | 494.556681 | 0 | 0.613688 | 90.218206 | 10.223073 |
| 22 | 0.157080 | -16.053927 | 0 | 0.131789 | 0.186185 | 483.223003 | 0 | 0.609048 | 90.010808 | 10.010820 |
| 23 | 0.160489 | -15.759681 | 0 | 0.134660 | 0.190183 | 489.563523 | 0 | 0.614974 | 90.166593 | 10.169415 |
| 24 | 0.157910 | -15.864789 | 0 | 0.132313 | 0.187388 | 494.022592 | 0 | 0.622312 | 90.245488 | 10.251666 |
| 25 | 0.160574 | -15.923026 | 0 | 0.134989 | 0.189943 | 492.709409 | 0 | 0.606875 | 90.123029 | 10.124562 |
| 26 | 0.161385 | -15.882915 | 0 | 0.135718 | 0.190834 | 490.335776 | 0 | 0.600909 | 90.022291 | 10.022340 |
| 27 | 0.160006 | -15.769901 | 0 | 0.134206 | 0.189679 | 493.261545 | 0 | 0.618506 | 90.249060 | 10.255421 |
| 28 | 0.160029 | -15.914741 | 0 | 0.134447 | 0.189415 | 494.328396 | 0 | 0.611538 | 90.191046 | 10.194767 |
| 29 | 0.159052 | -15.752821 | 0 | 0.133253 | 0.188758 | 495.040576 | 0 | 0.625360 | 90.283455 | 10.291725 |
| 30 | 0.159508 | -15.794510 | 0 | 0.133759 | 0.189131 | 494.912714 | 0 | 0.620773 | 90.297192 | 10.306295 |
| 31 | 0.156189 | -16.717661 | 0 | 0.131858 | 0.184058 | 461.673168 | 0 | 0.555174 | 89.221759 | 9.277952 |
| 32 | 0.160312 | -15.917136 | 0 | 0.134725 | 0.189693 | 493.556218 | 0 | 0.609292 | 90.158392 | 10.160942 |
| 33 | 0.158873 | -15.731318 | 0 | 0.133047 | 0.188622 | 477.425723 | 0 | 0.627278 | 86.814455 | 7.584062 |
| 34 | 0.157154 | -16.073506 | 0 | 0.131889 | 0.186221 | 489.669788 | 0 | 0.608302 | 90.093742 | 10.094629 |
| 35 | 0.163717 | -16.018595 | 0 | 0.138195 | 0.192898 | 433.286894 | 0 | 0.567023 | 89.312005 | 9.356292 |
| 36 | 0.158110 | -15.813870 | 0 | 0.132431 | 0.187690 | 494.167348 | 0 | 0.625020 | 90.111992 | 10.113260 |
| 37 | 0.160911 | -15.856841 | 0 | 0.135217 | 0.190411 | 491.942485 | 0 | 0.606767 | 90.109303 | 10.110511 |
| 38 | 0.158086 | -15.824972 | 0 | 0.132425 | 0.187644 | 494.463804 | 0 | 0.624437 | 90.205854 | 10.210181 |
| 39 | 0.159970 | -15.914813 | 0 | 0.134389 | 0.189354 | 494.471593 | 0 | 0.611960 | 90.197158 | 10.201124 |
| 40 | 0.159833 | -15.915797 | 0 | 0.134257 | 0.189216 | 494.770923 | 0 | 0.612847 | 90.210011 | 10.214516 |
| 41 | 0.159227 | -15.849788 | 0 | 0.133570 | 0.188739 | 495.464484 | 0 | 0.619624 | 90.296150 | 10.305188 |
| 42 | 0.159664 | -15.918687 | 0 | 0.134098 | 0.189041 | 495.083862 | 0 | 0.613787 | 90.223601 | 10.228715 |
| 43 | 0.158996 | -15.863128 | 0 | 0.133366 | 0.188481 | 495.590676 | 0 | 0.620031 | 90.302299 | 10.311722 |
| 44 | 0.158451 | -15.846987 | 0 | 0.132812 | 0.187966 | 495.502801 | 0 | 0.622671 | 90.320023 | 10.330603 |
| 45 | 0.159418 | -15.746797 | 0 | 0.133600 | 0.189137 | 494.161689 | 0 | 0.623652 | 90.279076 | 10.287088 |
| 46 | 0.157979 | -15.849929 | 0 | 0.132358 | 0.187487 | 494.248974 | 0 | 0.623129 | 90.242766 | 10.248806 |
| 47 | 0.158106 | -15.892503 | 0 | 0.132545 | 0.187530 | 495.005325 | 0 | 0.620738 | 90.294578 | 10.303519 |
| 48 | 0.158331 | -15.828379 | 0 | 0.132667 | 0.187883 | 495.402214 | 0 | 0.623914 | 90.310662 | 10.320622 |
| 49 | 0.156684 | -16.298699 | 0 | 0.131757 | 0.185320 | 484.323477 | 0 | 0.590722 | 89.840398 | 9.842905 |
| 50 | 0.156847 | -16.150739 | 0 | 0.131704 | 0.185764 | 479.526461 | 0 | 0.601509 | 89.919327 | 9.919973 |
| 51 | 0.156948 | -16.085762 | 0 | 0.131707 | 0.185990 | 466.639362 | 0 | 0.606261 | 89.799526 | 9.803466 |
| 52 | 0.156475 | -16.359437 | 0 | 0.131641 | 0.184996 | 463.741438 | 0 | 0.584276 | 89.630708 | 9.643860 |
| 53 | 0.160871 | -15.897526 | 0 | 0.135239 | 0.190291 | 492.237448 | 0 | 0.605361 | 90.096640 | 10.097584 |
| 54 | 0.161266 | -15.875559 | 0 | 0.135591 | 0.190729 | 490.760847 | 0 | 0.602454 | 90.045390 | 10.045597 |
| 55 | 0.159970 | -15.914813 | 0 | 0.134389 | 0.189354 | 494.471593 | 0 | 0.611960 | 90.197158 | 10.201124 |
| 56 | 0.157819 | -15.943831 | 0 | 0.132343 | 0.187141 | 494.231257 | 0 | 0.617863 | 90.255702 | 10.262412 |
| 57 | 0.159672 | -15.735050 | 0 | 0.133828 | 0.189415 | 492.444620 | 0 | 0.622581 | 90.232854 | 10.238405 |
| 58 | 0.159675 | -15.740581 | 0 | 0.133840 | 0.189407 | 492.975388 | 0 | 0.622294 | 90.251219 | 10.257692 |
| 59 | 0.158854 | -15.761827 | 0 | 0.133074 | 0.188541 | 495.410051 | 0 | 0.625798 | 90.284016 | 10.292318 |
| 60 | 0.160677 | -15.926233 | 0 | 0.135094 | 0.190040 | 492.342803 | 0 | 0.605842 | 90.107833 | 10.109008 |
| 61 | 0.158852 | -15.844188 | 0 | 0.133197 | 0.188375 | 495.606632 | 0 | 0.621579 | 90.317300 | 10.327698 |
| 62 | 0.161403 | -15.925867 | 0 | 0.135800 | 0.190767 | 490.401613 | 0 | 0.598910 | 90.000541 | 10.000541 |
| 63 | 0.157359 | -15.971245 | 0 | 0.131938 | 0.186626 | 488.436314 | 0 | 0.615262 | 90.110925 | 10.112169 |
| 64 | 0.157001 | -16.089114 | 0 | 0.131763 | 0.186037 | 483.395521 | 0 | 0.606427 | 89.998522 | 9.998522 |
| 65 | 0.160085 | -15.914861 | 0 | 0.134501 | 0.189470 | 494.189584 | 0 | 0.611131 | 90.185140 | 10.188633 |
| 66 | 0.162573 | -15.917276 | 0 | 0.136927 | 0.191953 | 478.436121 | 0 | 0.585863 | 89.749626 | 9.755741 |
| 67 | 0.159458 | -15.728428 | 0 | 0.133610 | 0.189213 | 492.411887 | 0 | 0.624308 | 90.191843 | 10.195595 |
| 68 | 0.157828 | -15.903728 | 0 | 0.132293 | 0.187229 | 494.090273 | 0 | 0.620112 | 90.261295 | 10.268306 |
| 69 | 0.162092 | -15.912610 | 0 | 0.136451 | 0.191482 | 486.662329 | 0 | 0.591857 | 89.876562 | 9.878067 |
| 70 | 0.159224 | -15.897594 | 0 | 0.133638 | 0.188641 | 495.537637 | 0 | 0.617256 | 90.269852 | 10.277336 |
| 71 | 0.156147 | -16.778116 | 0 | 0.131899 | 0.183909 | 453.986507 | 0 | 0.549944 | 89.112729 | 9.185038 |
| 72 | 0.161016 | -15.760601 | 0 | 0.135174 | 0.190709 | 474.324768 | 0 | 0.609929 | 89.964411 | 9.964537 |
| 73 | 0.158720 | -15.769207 | 0 | 0.132956 | 0.188392 | 495.557289 | 0 | 0.625940 | 90.280124 | 10.288197 |
| 74 | 0.160124 | -15.764402 | 0 | 0.134312 | 0.189808 | 492.533672 | 0 | 0.617838 | 90.231682 | 10.237177 |
| 75 | 0.160894 | -15.815415 | 0 | 0.135138 | 0.190477 | 490.911476 | 0 | 0.608745 | 90.122437 | 10.123955 |
| 76 | 0.159664 | -15.918687 | 0 | 0.134098 | 0.189041 | 495.083862 | 0 | 0.613787 | 90.223601 | 10.228715 |
| 77 | 0.160085 | -15.914861 | 0 | 0.134501 | 0.189470 | 494.189584 | 0 | 0.611131 | 90.185140 | 10.188633 |
| 78 | 0.157592 | -15.922112 | 0 | 0.132091 | 0.186956 | 491.779471 | 0 | 0.618750 | 90.187173 | 10.190743 |
| 79 | 0.160611 | -15.758369 | 0 | 0.134777 | 0.190308 | 487.760699 | 0 | 0.613926 | 90.135521 | 10.137383 |
| 80 | 0.160214 | -15.772712 | 0 | 0.134413 | 0.189882 | 492.534074 | 0 | 0.616725 | 90.223235 | 10.228332 |
| 81 | 0.158967 | -15.743335 | 0 | 0.133156 | 0.188691 | 494.845990 | 0 | 0.626245 | 90.202932 | 10.207136 |
| 82 | 0.157073 | -16.091517 | 0 | 0.131836 | 0.186104 | 488.235740 | 0 | 0.606746 | 90.060420 | 10.060788 |

**Supplementary Table 3. Results of univariate meta-regression.**

| Covariates | Tau^2^ | I^2^ (%) | R^2^ | Residual Heterogeneity | Test of Moderator |
| --- | --- | --- | --- | --- | --- |
| Income of Country or Area | 0 | 96.64 | 0 | <.01 | .9 |
| Development of Country | 0 | 96.64 | 0 | <.01 | .9 |
| Study Quality Score | 0 | 96.64 | 0 | <.01 | .9 |
| Study Size | 0 | 92.58 | .6 | <.01 | <.01 |
| Geographic Region | 0 | 96.91 | 0 | <.01 | .9 |
| Country or Area | 0 | 96.79 | 0 | <.01 | .8 |

**Supplementary Table 4. Definitions of surgical procedure.**

| Surgical procedure | Definition |
| --- | --- |
| PD | PD is a complex surgical procedure involving the removal of the head of the pancreas, duodenum, gallbladder, and a portion of the common bile duct. The remaining pancreas is typically reconnected to the digestive tract, while the bile duct and stomach are also reconstructed. |
| DP | DP is the surgical removal of the tail of the pancreas, which may also include the body of the pancreas depending on the extent of the disease. The remaining pancreas is connected to the digestive tract. |
| DPPHR | DPPHR is a surgical procedure involving the resection of a portion of the pancreatic head while preserving both the duodenum and a significant portion of the remaining pancreas including the pancreatic isthmus. |
| PHRSD | PHRSD involves the removal of a segment of the duodenum along with the pancreatic head. The procedure is designed to address conditions related to the pancreatic head while preserving the pancreatic isthmus and the majority of the pancreas. |
| CP | CP involves the removal of the central portion of the pancreas, sparing the head and tail. The cut ends of the pancreas are then meticulously sutured or stapled. CP is performed when the lesion or disease is localized to the central pancreas while preserving the adjacent pancreatic isthmus. |
| TEU | TEU is a minimally invasive technique that involves the careful removal of tumors from the pancreas, leaving the majority of the organ intact. It requires precise dissection to separate the tumor from surrounding tissues. |

PD, pancreatoduodenectomy; DP, distal pancreatectomy; DPPHR, duodenum-preserving pancreatic head resection; PHRSD, pancreatic head resection with segmental duodenectomy; CP, central pancreatectomy; TEU, tumor enucleation

**Supplementary Figure 1. Leave-one-out diagnostics with a built-in function in metafor.**


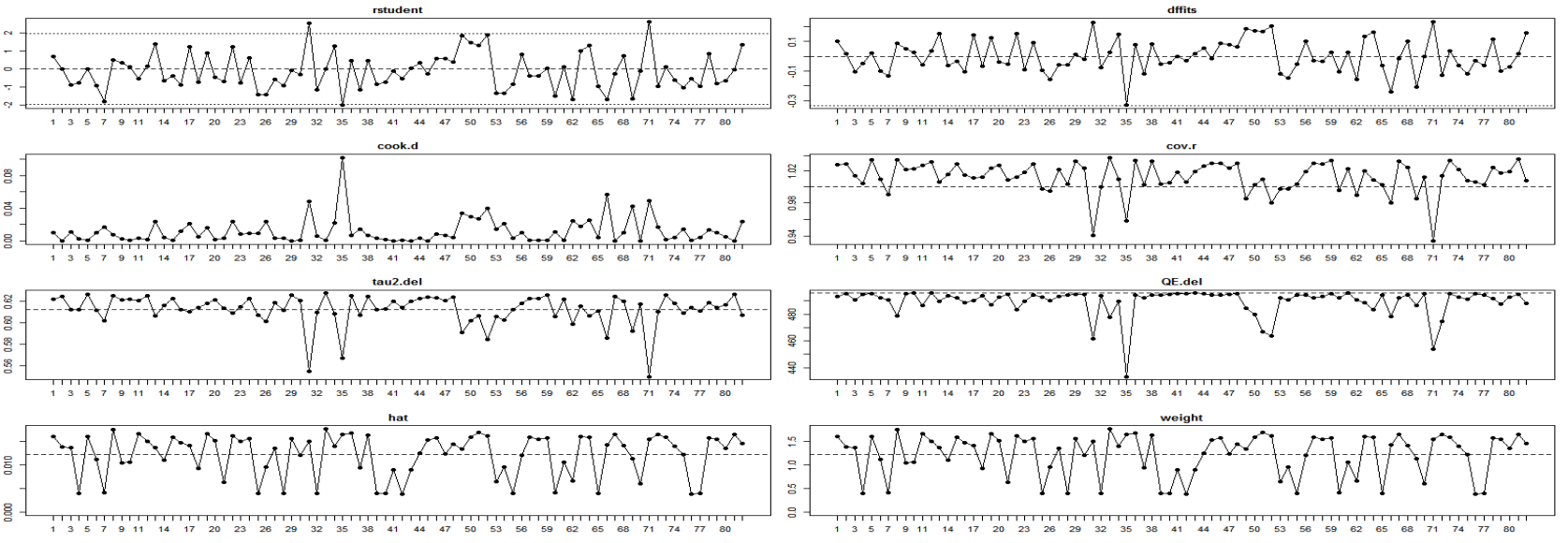


**Supplemetary Figure 2. Utilization of insulin in patients developing new-onset diabetes.**


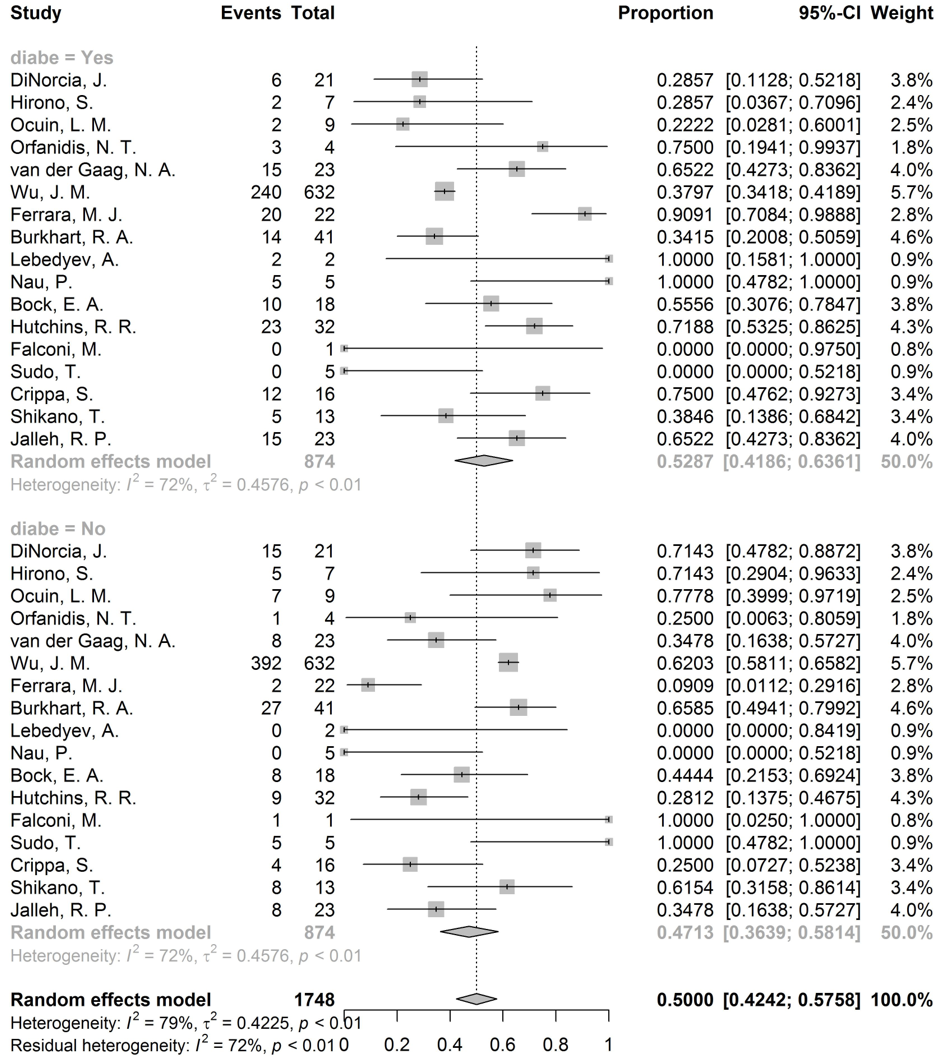


**Supplementary Figure 3. Pooled prevalence of worsening and resolution of diabetes in patients with preoperative diabetes.**

**(A) Pooled prevalence of worsening diabetes in patients with preoperative diabetes.**

**
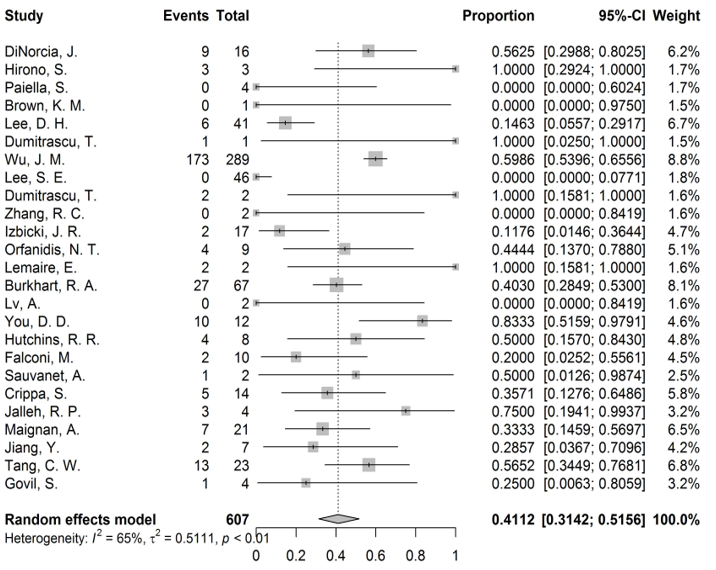
**

**(B) Pooled prevalence of resolution of diabetes in patients with preoperative diabetes.**


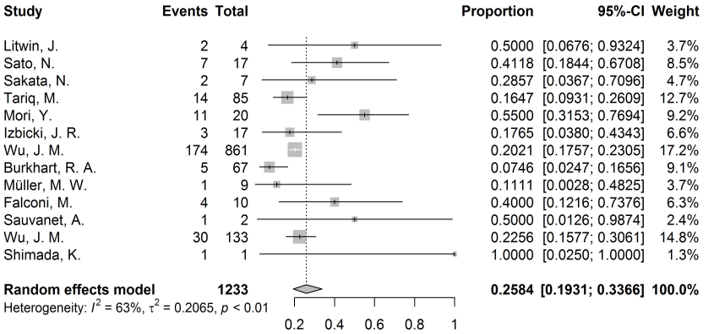

Supplement: SUPPLEMENTARY MATERIAL [file js9-110-1770-s004.docx]
